# Tools for Genetic Studies in Experimental Populations of Polyploids

**DOI:** 10.3389/fpls.2018.00513

**Published:** 2018-04-18

**Authors:** Peter M. Bourke, Roeland E. Voorrips, Richard G. F. Visser, Chris Maliepaard

**Affiliations:** Plant Breeding, Wageningen University & Research, Wageningen, Netherlands

**Keywords:** polyploid genetics, polyploid software tools, autopolyploid, allopolyploid, segmental allopolyploid

## Abstract

Polyploid organisms carry more than two copies of each chromosome, a condition rarely tolerated in animals but which occurs relatively frequently in the plant kingdom. One of the principal challenges faced by polyploid organisms is to evolve stable meiotic mechanisms to faithfully transmit genetic information to the next generation upon which the study of inheritance is based. In this review we look at the tools available to the research community to better understand polyploid inheritance, many of which have only recently been developed. Most of these tools are intended for experimental populations (rather than natural populations), facilitating genomics-assisted crop improvement and plant breeding. This is hardly surprising given that a large proportion of domesticated plant species are polyploid. We focus on three main areas: (1) polyploid genotyping; (2) genetic and physical mapping; and (3) quantitative trait analysis and genomic selection. We also briefly review some miscellaneous topics such as the mode of inheritance and the availability of polyploid simulation software. The current polyploid analytic toolbox includes software for assigning marker genotypes (and in particular, estimating the dosage of marker alleles in the heterozygous condition), establishing chromosome-scale linkage phase among marker alleles, constructing (short-range) haplotypes, generating linkage maps, performing genome-wide association studies (GWAS) and quantitative trait locus (QTL) analyses, and simulating polyploid populations. These tools can also help elucidate the mode of inheritance (disomic, polysomic or a mixture of both as in segmental allopolyploids) or reveal whether double reduction and multivalent chromosomal pairing occur. An increasing number of polyploids (or associated diploids) are being sequenced, leading to publicly available reference genome assemblies. Much work remains in order to keep pace with developments in genomic technologies. However, such technologies also offer the promise of understanding polyploid genomes at a level which hitherto has remained elusive.

## Introduction

One of the most fundamental descriptions of any organism is its ploidy level and chromosome number, generally written in the form 2*n* = 2*x* = 10 (here, for the ubiquitous model plant species *Arabidopsis thaliana* L.). Plant scientists in particular will be familiar with this representation of the chromosomal constitution of the sporophyte generation (i.e., the adult plant). The second term in this seemingly simple equation describes the normal complement of chromosomal copies possessed by a member of that species, which is generally 2× (“two times”) for diploids. Species where this number exceeds two are collectively referred to as polyploids. Not unexpectedly, each polyploid individual is the product of the fusion of gametes from two parents, just like their diploid counterparts. In other words, polyploids can also be defined as individuals derived from non-haploid gametes (in the case of triploids derived from diploid × tetraploid crosses, only one gamete satisfies this condition). The transmission of non-haploid gametes is one of the main “complexifying” features of polyploidy, leading to a whole range of implications for the genetic analysis of these “hopeful monsters” (Goldschmidt, 1933).

The ongoing genomics revolution can be seen as a rising tide which has also lifted the polyploid genetics boat, although not quite to the same level as for diploids. Most genetic advances are made in model organisms, among which self-fertilizing diploid species predominate. It is therefore not surprising that most tools and techniques for molecular-genetic studies are specific to diploids. However, polyploid species are particularly important to mankind in the provision of food, fuel, feed, and fiber (not to mention “flowers,” if ornamental plant species are also included), making the genetic analysis of polyploid species an important avenue of research for crop improvement.

Although a collective term such as “polyploidy” has its uses, it tends to obscure some fundamental differences between its members. For example, polyploids are generally subdivided into autopolyploids and allopolyploids (Kihara and Ono, 1926). Autopolyploids arise through genomic duplication within a single species, generally through the production of unreduced gametes (Harlan and De Wet, 1975) and exhibit polysomic inheritance, meaning pairing and recombination can occur between all homologous copies of each chromosome during meiosis. One of the most well-studied examples is autotetraploid potato (*Solanum tuberosum* L.). Allopolyploids, on the other hand, are the product of genomic duplication between species [usually through hybridisation involving unreduced gametes (Harlan and De Wet, 1975)] and display disomic inheritance, where more-related chromosome copies (“homologs”) may pair and recombine during meiosis, whilst less-related chromosome copies [“homoeologs,” also spelled “homeologs” (Glover et al., 2016)] do not. Among allopolyploids, allohexaploid wheat (*Triticum aestivum* L.) is probably the most well-studied. If pairing and recombination between homoeologs occurs to a limited extent, the species may be referred to as “segmental allopolyploid” (Stebbins, 1947), traditionally deemed to have arisen from hybridisation between very closely related species (Stebbins, 1947; Chester et al., 2012) but which may also be the result of partially diploidised autopolyploidy (Soltis et al., 2016). In many cases, a species cannot be clearly designated as one type or another, leading to uncertainty or debate on the subject (Barker et al., 2016; Doyle and Sherman-Broyles, 2016). From the perspective of genetics and inheritance, allopolyploids behave much like diploid species and therefore many of the tools developed for diploids can be directly applied. The main challenge that faces allopolyploid geneticists is in distinguishing between homoeologous gene copies carried by sub-genomes within an individual (Kaur et al., 2012; van Dijk et al., 2012; Rothfels et al., 2017). Autopolyploids (and segmental allopolyploids) do not behave like diploids, and are therefore in most need of specialized methods and tools for subsequent genetic studies. In this review we focus primarily on the availability of tools and resources amenable to polysomic [and “mixosomic” (Soltis et al., 2016)] species, with less emphasis on allopolyploid-specific solutions. Although the development of novel methodologies for the genetic analysis of polyploids are interesting, without translation into a software tool for use by the research community they remain purely conceptual and with limited impact. We therefore try to limit our attention to the tools currently available rather than cataloging descriptions of unimplemented methods.

Experimental populations, in use since Mendel’s ground-breaking work (Mendel, 1866), are traditionally derived from a controlled cross between two parental lines of interest (either directly studying the F_1_ or some later generation). We use the term here to distinguish our subject matter from “wild” or “natural” populations, which would necessitate sampling individuals from an extant population in the wild. Quantitative genetics, particularly the genetics of human pathology, has greatly benefitted from the use of large panels of individuals to perform so-called “genome-wide association studies” (GWAS). The use of such panels offers to complement the experimental toolbox of polyploid geneticists as well, and although perhaps not strictly speaking an “experimental” population, we consider them relevant to the current discussion.

Here, we review three main areas: (1) polyploid genotyping, including the scoring of marker dosage (allele counts) and generation of haplotypes; (2) genetic and physical mapping, where we look at the possibilities for linkage mapping as well as the availability of reference sequences; and (3) quantitative trait analysis and genomic selection, including tools that perform quantitative trait locus (QTL) analysis in bi-parental populations, genome-wide association analysis (GWAS) and genomic selection and prediction. We also consider the current tools to simulate polyploid organisms for *in silico* studies, as well as those that can help determine the mode of inheritance of the species being studied. We reflect on current and future developments, and the tools that will be needed to keep pace with the innovations we are witnessing in genomic technologies.

## Polyploid Genotyping

One of the most crucial aspects in the study of polyploid genetics is the generation of accurate genotypic data. However, it is also fraught with difficulties, not least the detection of multiple loci when only a single locus is targeted (Mason, 2015; Limborg et al., 2016). Various technologies exist, with almost all current applications aimed at identifying single nucleotide polymorphisms (SNPs). Although many genomic “service-providers” (e.g., companies or institutes that offer DNA sequencing) have their own tools to analyze and interpret raw data, these tools are not always suitable for use with polyploid datasets. Gel-based marker technologies continue to be used and retain certain advantages (e.g., low costs associated with small marker numbers, requiring only basic laboratory facilities, multi-allelism etc.). However, most studies now rely on SNP markers for genotyping due to their great abundance over the genome, their high-throughput capacity and their low cost per data point. Targeted genotyping such as SNP arrays (a.k.a. “SNP chips”) rely on previously identified and selected polymorphisms, usually identified from a panel of individuals chosen to represent the gene pool under investigation. In contrast, untargeted genotyping generally uses direct sequencing of individuals, albeit after some procedure to reduce the amount of DNA to be sequenced [e.g., by exome sequencing (Ng et al., 2009) or target enrichment (Mamanova et al., 2010)]. The disadvantages of targeted approaches have been well explored (particularly regarding ascertainment bias, where the set of targeted SNPs on an array poorly represents the diversity in the samples under investigation due to biased methods of SNP discovery) (Albrechtsen et al., 2010; Moragues et al., 2010; Didion et al., 2012; Lachance and Tishkoff, 2013), although there are advantages and disadvantages to both methods (Mason et al., 2017). Apart from costs, differences exist in the ease of data analysis following genotyping, with sequencing data requiring greater curation and bioinformatics skills (Spindel et al., 2013; Bajgain et al., 2016) as well as potentially containing more erroneous and missing data (Spindel et al., 2013; Jones et al., 2017).

In polyploids, SNP arrays have been developed in numerous species [recently reviewed by (You et al., 2018)], which include both autopolyploid (or predominantly polysomic polyploids) and allopolyploid species. Examples of the former include alfalfa (Li et al., 2014), chrysanthemum (van Geest et al., 2017b), potato (Hamilton et al., 2011; Felcher et al., 2012; Vos et al., 2015), rose (Koning-Boucoiran et al., 2015) and sour cherry (Peace et al., 2012). Examples of allopolyploid SNP arrays include cotton (Hulse-Kemp et al., 2015), oat (Tinker et al., 2014), oilseed rape (Dalton-Morgan et al., 2014; Clarke et al., 2016), peanut (Pandey et al., 2017), strawberry (Bassil et al., 2015) and wheat (Akhunov et al., 2009; Cavanagh et al., 2013; Wang et al., 2014; Winfield et al., 2016). Untargeted approaches such as genotyping using next-generation sequencing have also been applied, for example in autopolyploids such as alfalfa (Zhang et al., 2015; Yu et al., 2017), blueberry (McCallum et al., 2016), bluestem prairie grass (*Andropogon gerardii*) (McAllister and Miller, 2016), cocksfoot (*Dactylis glomerata*) (Bushman et al., 2016), potato (Uitdewilligen et al., 2013; Sverrisdóttir et al., 2017), sugarcane (Balsalobre et al., 2017; Yang et al., 2017b) and sweet potato (Shirasawa et al., 2017), and in allopolyploids such as coffee (Moncada et al., 2016), cotton (Islam et al., 2015; Reddy et al., 2017), intermediate wheatgrass (*Thinopyrum intermedium*) (Kantarski et al., 2017), oat (Chaffin et al., 2016), prairie cordgrass (*Spartina pectinata*) (Crawford et al., 2016), shepherd’s purse (*Capsella bursa-pastoris*) (Cornille et al., 2016), wheat (Poland et al., 2012; Edae et al., 2015), and zoysiagrass (*Zoysia japonica*) (McCamy et al., 2018) (noting that the precise classification of some of these species as auto- or allopolyploids has yet to be conclusively determined). Whatever the technology used, it is clear that we are currently witnessing an explosion of interest in polyploid genomics. However, the critical issue of how to make sense of this data remains, starting with the assignment of marker dosage, a.k.a. “genotype calling.”

### Assignment of Dosage

**FIGURE 1:**
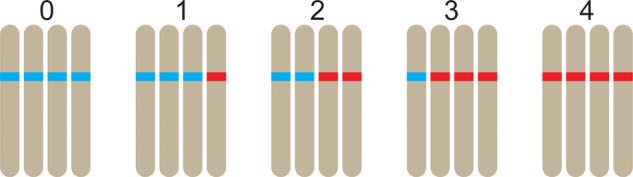
In a tetraploid, five distinct dosages are possible at a bi-allelic marker positions, ranging from 0 copies of the alternative allele through to 4 copies. Here, the alternative allele is colored red, with the reference allele colored blue.

One of the key distinguishing features of polysomic polyploidy is the fact that there are multiple heterozygous conditions possible in genotyping data. We use the term marker “dosage” to denote the minor allele count of a marker; a species of ploidy *q* possesses *q* + 1 distinct dosage classes in the range 0 to *q* (**Figure 1**). Of course the concept of marker dosage could also be used in diploid species, but coding systems such as the lm × ll / nn × np / hk × hk system (Van Ooijen, 2006) predominate. Marker dosage is generally understood to apply to bi-allelic markers (such as single SNPs), although it is conceivable to score marker dosage at multi-allelic loci. If marker dosage cannot be accurately assessed, genotypes would likely have to be dominantly scored (i.e., all heterozygous classes would be grouped with one of the homozygous classes), resulting in a loss of information (Piepho and Koch, 2000).

All available dosage-calling tools rely on a population in order to determine marker dosage. In other words, calibration between the various dosage classes is performed across the population (for which we are not implying any degree of relatedness in the population other than coming from the same species). All current tools are designed to process genotyping data from SNP arrays, using the relative strength of two allele-specific (fluorescent) signals to assign a discrete dosage value. With increasing interest in genotyping using next generation sequencing (GNGS), we anticipate that tools which use read-counts of potentially multiple SNPs (or multi-SNP haplotypes) will soon be developed, although these have yet to appear. One of the current challenges under investigation regarding GNGS-based genotype calling is the accurate determination of dosage (Kim et al., 2016), which may require relatively deep sequencing [e.g., 60–80 × coverage estimated in autotetraploid potato (Uitdewilligen et al., 2013)].

Returning to the SNP array-based tools, the two main service providers for high-density SNP arrays, Illumina and Affymetrix, both offer proprietary software solutions for analyzing polyploid datasets. Affymetrix’s Power Tools and Illumina’s GenomeStudio (with its Polyploid Genotyping Module) have both been developed with both diploid and polyploid datasets in mind. However, there have also been a number of genotyping tools that have been put into the public domain. One of the first of these to be released was fitTetra (Voorrips et al., 2011), a freely available R package (R Core Team, 2016) designed to assign genotypes to autotetraploids that were genotyped on either Illumina’s Infinium or Affymetrix’s Axiom arrays. fitTetra fits mixture models to bi-allelic SNP intensity ratios either under the constraint of Hardy-Weinberg equilibrium within the population, or as an unconstrained fit, using an expectation-maximization (EM) algorithm in fitting. This can have the drawback of requiring significant computational resources for high-density marker datasets, although it is automated and can therefore process large datasets in a single run. The original release was specific to tetraploid data only. However, an updated version (fitPoly) can process genotyping data of all ploidy levels and has recently made available as a separate R package on CRAN^1^. The SuperMASSA application (Serang et al., 2012) can also process data from all ploidy levels (as it was initially developed to dosage-score sugarcane data, notorious for its cytogenetic complexity) and is currently hosted online by the Statistical Genetics Laboratory in the University of São Paulo, Brazil. One of the interesting features of SuperMASSA is that prior knowledge of the exact ploidy level is not needed (useful for a crop like sugarcane). Instead, the genotype configuration which maximizes the posterior probability across all specified ploidy levels is chosen. In practice, most researchers will already know the ploidy of their samples (although aneuploid progeny in some species may occur) and can constrain the model search. A draw-back of the online implementation is that markers are analyzed one-by-one, and results need to be copied from the webpage each time. However, a command-line version of SuperMASSA is currently under development.

The R package polysegRatioMM (Baker et al., 2010) generates marker dosages for dominantly scored markers using the JAGS software (Plummer, 2003) for Markov Chain Monte Carlo (MCMC) generation. Fully polysomic behavior is assumed, and segregation ratios of marker data are used to derive the most likely parental scores. Although able to process data from all even ploidy levels, the software only considers a subset of marker types (marker that are nulliplex in one parent or simplex in both parents). Nowadays, there is a move away from dominantly scored markers to co-dominant marker technologies like SNPs, and parental samples are usually included in multiple replicates (and so can be genotyped directly with offspring, rather than imputed from the offspring). The package is therefore of questionable use for modern genotyping datasets. An unrelated R package, beadarrayMSV (Gidskehaug et al., 2010), was developed to handle Illumina Infinium SNP array data from “diploidising” tetraploid species such as the Atlantic salmon. The software was designed to score markers which target multiple loci (so-called multi-site variants, or MSVs), as well as single-locus markers displaying disomic inheritance. In a comparison with fitTetra, beadarrayMSV was unable to accurately genotype autotetraploid data from potato, although conversely fitTetra performed poorly on salmon data (Voorrips et al., 2011). This demonstrates that appropriate software is needed for specific situations (indeed, in many cases specific scenarios have motivated the development of specialized software).

Having prior knowledge about the expected meiotic behavior of the species is always advantageous when it comes to analyzing any polyploid data. This is especially true for the latest dosage-calling software to be released, the ClusterCall package for R (Schmitz Carley et al., 2017). Here, prior knowledge of the meiotic behavior of the species is required, since the expected segregation ratios of an F_1_ autotetraploid population are used to assign dosage scores to the clusters identified through hierarchical clustering. In well-behaved autotetraploids such as potato (Swaminathan and Howard, 1953; Bourke et al., 2015) this is arguably not a problem (as long as skewed segregation does not occur), and indeed can lead to increased accuracy in genotype calling (Schmitz Carley et al., 2017). However, in less well-characterized species such as leek, alfalfa, or many ornamental species, the precise meiotic behavior may not always follow the expected tetrasomic model, causing potential problems with fitting. The authors are aware of this and suggest that alternatives like fitTetra or SuperMASSA be used in circumstances where a tetrasomic model no longer holds. Unfortunately, such prior knowledge is not always available before genotyping takes place – meiotic behavior can even differ between individuals of a species that was thought to display meiotic homogeneity (e.g., complete tetrasomy) (Bourke et al., 2017).

### Haplotype Assembly

**FIGURE 2:**
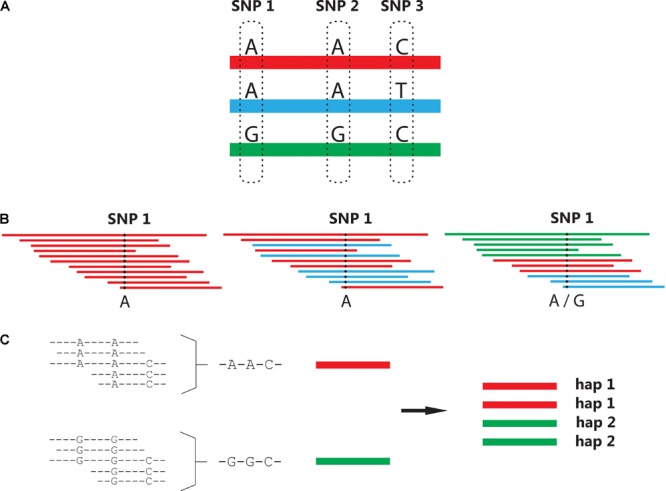
Generation of multi-SNP haplotypes. **(A)** In this example, three possible haplotypes exist spanning polymorphic positions SNP 1, 2, and 3. **(B)** Single-SNP genotyping cannot distinguish between the “A” allele originating from different haplotypes, combining them into a single allele as illustrated in the second SNP call. **(C)** In a haplotyping approach, overlapping reads are used to re-assemble and phase single SNP genotypes. Here, the known ploidy level of the species (4×) is used to impute the dosage of the two haplotypes identified in this individual, given a 1:1 ratio between the assembled haplotype read-depths.

Although bi-allelic SNP markers have many practical advantages, they carry less inheritance information than multi-allelic markers. Crop researchers and breeders often wish to develop a simple diagnostic marker test for a trait of interest. Unfortunately, the chances of having a single SNP in complete linkage disequilibrium with a favorable or causative allele of a gene of interest is very small. Markers which have been found to uniquely “tag” a favorable allele in one population may not do so in another. For more than a decade, the increased power of haplotype-based associations have been known and reported in human genetic studies (Zhang et al., 2002; de Bakker et al., 2005), with the term “haplotype” denoting a unique stretch of sequence. Translating haplotyping approaches from diploid to polyploid species has been a non-trivial exercise, requiring novel algorithms to handle the overwhelming range of possibilities that can arise [especially when allowing for sequencing errors and (possible) recombinations]. Multi-SNP haplotypes can be assembled from single dosage-scored SNPs (originating from SNP array data), although haplotypes are more commonly generated using overlapping sequence reads (**Figure 2**).

A number of different polyploid haplotyping tools (for sequence reads) have been developed in recent years, including polyHap (Su et al., 2008), SATlotyper (Neigenfind et al., 2008), HapCompass (Aguiar and Istrail, 2013), HapTree (Berger et al., 2014), SDhaP (Das and Vikalo, 2015), SHEsisplus (Shen et al., 2016), and TriPoly (Motazedi et al., unpublished). Three of these tools (HapCompass, HapTree, and SDhaP) were recently compared and evaluated over a range of different simulated read depths, ploidy levels and insert sizes for paired-end reads (Motazedi et al., 2017). The authors found that each of these software programs had particular advantages, for example HapTree was found to produce more accurate haplotypes for triploid and tetraploid data, whilst HapCompass performed best at higher ploidies (6× and higher) (Motazedi et al., 2017). Both SHEsisplus and TriPoly have yet to be independently tested. For allopolyploid species, the user-friendly Haplotag software has been designed to identify both single SNPs and multi-SNP haplotypes from genotypes developed using next generation sequencing data (Tinker et al., 2016). An interesting feature is the use of a simple “heterozygosity filter” that excludes haplotypes with higher than expected heterozygosity across a population (suggesting paralogous loci). Currently, however, data from outcrossing or autopolyploid species is not suitable for this software.

The input data of haplotyping software can be grouped into two types. Individual SNP genotyping data (with a known marker order) was used by the first wave of polyploid haplotyping implementations such as polyHap and SATlotyper. More recently, haplotyping tools use sequence reads as their input, although some pre-processing is required: reads must first be aligned followed by extraction of their SNPs (i.e., masking of non-polymorphic sites) to generate a SNP-fragment matrix with individual reads as rows and SNP positions as columns [as described for HapCompass (Aguiar and Istrail, 2013)]. In other words, all haplotyping tools [apart perhaps from Haplotag (Tinker et al., 2016)] require that users possess a certain level of bioinformatics skills. Although we expect polyploid haplotypes to become increasingly used in the future, the development of user-friendly and computationally efficient tools is first needed before haplotype-based genotypes become truly mainstream.

One interesting development is the application of haplotyping to whole genome assemblies (as opposed to genotyping a population). This has recently been attempted in the tuberous hexaploid crop sweet potato (*Ipomoea batatas*) (Yang et al., 2017a). The authors first produced a consensus assembly to which reads were re-mapped for variant calling, followed by a phasing algorithm which resolved the six haplotypes of the sequenced cultivar for about 30% of the assembly (Yang et al., 2017a). Ultimately, about half of the assembled genome could be haplotype-resolved. Future sequencing (or re-sequencing) efforts in polyploid species should produce more phased genomes, which will no doubt be useful for haplotyping applications (for example in validating predicted haplotypes).

## Genetic and Physical Mapping of Polyploid Genomes

One of the first steps in understanding the genetic composition of any species is the development of a map, be it a genetic map based on information about linkage and co-inheritance of specific DNA locations, or a physical map giving a reference DNA sequence for the species. In polyploid species, numerous technical and methodological complications arise that make the mapping of polyploids a much more complex endeavor than diploid mapping. However, there is currently an upsurge in interest in polyploid mapping, which has led to much progress in recent years.

### Linkage Maps

Although the first genetic linkage map was developed more than 100 years ago (Sturtevant, 1913), their use in genetic and genomic studies has persisted into the “next-generation” era. This can be attributed to a number of factors. A linkage map is a description of the recombination landscape within a species, usually from a single experimental cross of interest. For breeders, knowledge of genetic distance is arguably more important than physical distance, as it reflects the recombination frequencies in inheritance studies as well as describing the extent of linkage drag around loci of interest. Many software for performing QTL analysis require linkage maps of the markers, not physical maps. This is because co-inheritance of markers and phenotypes within a population are assumed to be coupled – a physical map gives less precise information about the co-inheritance of markers than a linkage map does since physical distances do not directly translate to recombination frequencies (particularly in the pericentromeric regions). Another reason why linkage maps continue to be developed is that they are often the first genomic representation of a species, upon which more advanced representations can be built. They provide useful long-range linkage information over the whole chromosome which is often missing from assemblies of short sequence reads. This fact has been repeatedly exploited in efforts at connecting and correctly orientating scaffolds during genome assembly projects (Bartholomé et al., 2015; Fierst, 2015).

**FIGURE 3:**
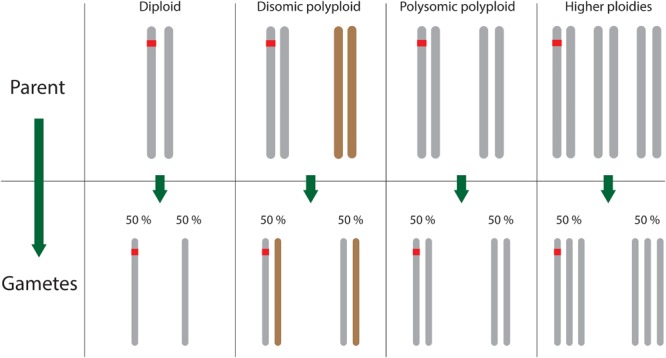
Simplex markers (carrying a single copy of the segregating marker allele) inherit similarly across all ploidy levels and pairing behaviors, allowing diploid mapping software to be used. Here, the (simplex) SNP allele is colored red.

As mentioned in the Introduction, polyploids can be divided into disomic or polysomic species, with the additional possibility of a mixture of both inheritance types in the case of segmental allopolyploids. Many linkage maps in polyploids have been based exclusively on 1:1 segregating markers, also known as simplex markers [because the segregating allele is in simplex condition (one copy) in one of the parents only]. These markers possess a number of advantages over other marker segregation types, but also some distinct disadvantages. In their favor, coupling-phase simplex markers in polyploid species behave just like they would in diploid species, regardless of the mode of inheritance involved (repulsion-phase recombination frequency estimates are not invariant across ploidy levels or modes of inheritance, but exert less influence on map construction due to lower LOD scores). The advantage of this is clear: in unexplored polyploid species for which the mode of inheritance is uncertain, simplex markers allow an “assumption-free” linkage map to be created, following which the mode of inheritance can be further explored. The only exception to this is if double reduction occurs, i.e., when a segment of a single chromosome gets transmitted with its sister chromatid copy to an offspring, a consequence of multivalent pairing and a particular sequence of segregation and division during meiosis (Haldane, 1930; Mather, 1935). Double reduction occurs randomly in polysomic species and only introduces a small bias into recombination frequency estimates (Bourke et al., 2015). This means that, ignoring the possible influence of double reduction, diploid mapping software can generally be used for simplex marker sets at any ploidy level and for any type of meiotic pairing behavior (**Figure 3**), opening up a very wide range of diploid-specific software options (Cheema and Dicks, 2009).

However, simplex marker sets have some limitations. Firstly, in selecting only simplex markers, a large proportion of markers with different segregation patterns are not used. This usually reduces the map coverage (while increasing the per-marker costs of the final set of mapped markers). More importantly, simplex markers give limited information about linkage in repulsion phase, particularly at higher ploidy levels (van Geest et al., 2017a). This means that homolog-specific maps can be produced, but they are unlikely to be well-integrated between homologs in a single parent, and impossible to integrate across parents. In other words, the chromosomal numbering will most likely be inconsistent between parental maps if only simplex markers are used. Producing a consensus or fully integrated map is desirable for many reasons, including being able to detect and model more complex QTL configurations than just simplex QTL. Therefore, a truly polyploid linkage mapping tool should be able to include all marker segregation types, not just 1:1 segregating markers.

### Polyploid Linkage Mapping Software

Linkage mapping can be broken into three steps – linkage analysis, marker clustering and marker ordering. There are still relatively few software tools that can perform all three of these steps for polysomic species. Perhaps the most well-known and widely used software tool is TetraploidMap for Windows (Hackett and Luo, 2003; Hackett et al., 2007). As well as producing linkage maps for autotetraploid species, this software also performs QTL interval mapping (returned to later). Recently, TetraploidMap was updated to enable the use of dosage-scored SNP data (Hackett et al., 2013). The updated version, TetraploidSNPMap (Hackett et al., 2017), is freely available to download from the Scottish BioSS website^2^, and possesses a sophisticated graphical user interface (GUI) which will be extremely welcome for users in both the research and breeding community. Apart from its dependency on the Windows platform, the main drawback of TetraploidSNPMap (TSNPM) is that it is programmed to analyze autotetraploid data only, and there is no indication when or if it will be expanded to other ploidy levels or modes of inheritance. However, tetraploidy is the most common polyploid condition (Comai, 2005) and therefore this software is still relevant for a broad range of species.

Recently, an alternative linkage mapping package called polymapR was released, which is described in a pre-print manuscript (Bourke et al., unpublished). Like TSNPM, polymapR used dosage-scored marker information from F_1_ populations to estimate recombination frequencies by maximum likelihood in a two-point linkage analysis. It can perform linkage analysis for polysomic triploids, tetraploids and hexaploids as well as segmental allotetraploid populations. As an R-based package it requires some level of user familiarity with R, but comes with a descriptive vignette which should make it accessible even to novice R users. It uses the same high-speed map ordering algorithm as TSNPM, namely MDSMap (Preedy and Hackett, 2016), and produces both integrated and phased linkage maps (i.e., separate maps for each parental homolog that are also integrated into a single consensus map). So far, developmental versions of this software have been used to generate high-density linkage maps in tetraploid potato (Bourke et al., 2016), tetraploid rose (Bourke et al., 2017), and hexaploid chrysanthemum (van Geest et al., 2017a).

Another recently released R package that can perform linkage map construction is the netgwas package, also described in a pre-print manuscript (Behrouzi and Wit, 2017a). netgwas claims to be able to construct maps at any ploidy level in both inbred and outbred bi-parental populations, and rather than computing recombination frequencies and LOD scores, it uses conditional dependence relationships between markers based on discrete graphical models. The algorithm automatically detects linkage groups (which are traditionally identified by a user-specified LOD threshold) and does not rely on knowledge of parental dosage scores (which should offer robustness against parental genotyping errors). The output of netgwas is clustered and ordered marker names, but without assigning genetic positions (centiMorgans) or marker phasing, which are part of the TSNPM and polymapR output. The lack of marker phasing in particular is a major drawback, as phase considerations are crucial in polyploid genetic analyses. However, given its novel and computationally efficient approach to map construction, it appears to be a very interesting addition to the current range of polyploid mapping tools.

**FIGURE 4:**
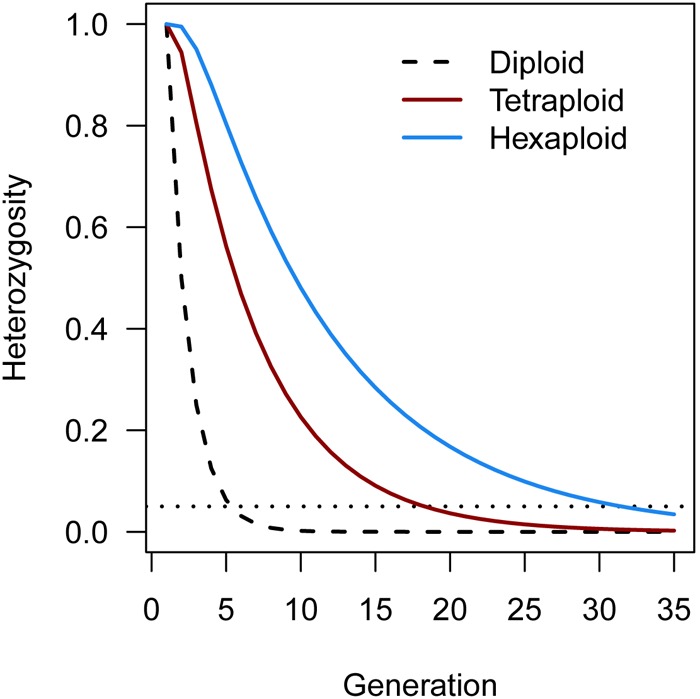
Theoretical rate of decrease in heterozygosity in polyploid species from repeated rounds of inbreeding/selfing, using expressions derived by Haldane (1930). For autotetraploids (red line), 95% homozygosity (horizontal dotted line) is achieved after on average 19 generations of selfing, while for a hexaploid (blue line) 95% homozygosity is reached after approximately 32 generations. By contrast, a diploid reaches 95% homozygosity after approximately 5 generations of selfing (black dashed line).

Another software program that is able to perform all three major steps in polyploid linkage mapping is the PERGOLA package in R (Grandke et al., 2017). This software can analyze marker data from all ploidy levels and modes of inheritance, but is limited to populations derived from completely inbred (homozygous) founder parents, such as F_2_ or BC_1_ populations. While these sorts of experimental population are common in diploid plant species, they are much less common in polyploids due to the difficulty in reaching homozygosity through selfing (Haldane, 1930). Generally speaking, polyploids are more heterozygous than diploids (Soltis and Soltis, 2000) although there is no general consensus regarding their tolerance of inbreeding (Krebs and Hancock, 1990; Soltis and Soltis, 2000; Galloway et al., 2003; Galloway and Etterson, 2007). There are indications that polyploid plant species self-fertilize more often than their diploid relatives (Barringer, 2007). However, regardless of whether polyploids tolerate some levels of inbreeding or not, heterozygosity is maintained for many more generations in repeatedly selfed polyploids than in selfed diploids (**Figure 4**). It therefore appears likely that PERGOLA was developed for newly formed polyploids derived from inbred diploid lines. The complexities facing extant (or heterozygous) polyploid species such as unknown marker phasing, or variable marker information contents are ignored by PERGOLA, making it doubtful that this tool will have a wide impact on linkage mapping in existing polyploid populations.

One final software that should be mentioned is PolyGembler, recently described in a pre-print manuscript (Zhou et al., unpublished). It proposes a novel approach to the creation of linkage maps in outcrossing polyploids, and is also suitable for diploid mapping. Interestingly, it combines a haplotyping algorithm [derived from the polyHap algorithm (Su et al., 2008)] to first generate phased multi-marker scaffolds or haplotypes. These are then used to calculate recombination frequencies by counting recombination events both within and between these scaffolds, leading to an extremely simple estimate of *r* which has no corresponding LOD score. Scaffolds are clustered using a graph partitioning algorithm, and thereafter, the computationally efficient CONCORDE traveling-salesman solver is employed to order markers [as is done for example in TSPmap (Monroe et al., 2017)]. This assumes that the variance of all *r* estimates is equal and that weights are not required – which may well be the case if the haplotype scaffolds are correctly constructed. PolyGembler claims to be able to handle the high levels of missing data and genotyping errors associated with next-generation sequencing data. Although it is applicable to multiple ploidy levels, the authors point out that mapping at the hexaploid level becomes computationally difficult due to the huge number of possible combinations in the formation of haplotypes. However, it appears to be a very promising tool which combines both genetic and bioinformatic approaches in a single pipeline.

Apart from those tools which constitute a complete linkage mapping pipeline, there have been some specific tools recently developed which we predict will have an important impact on future polyploid mapping applications. One of the most significant of these is the MDSMap package in R (Preedy and Hackett, 2016), a novel approach for determining a map order using multi-dimensional scaling. Marker data in polyploid species possesses variable information content, a fact that can be appreciated by considering the haplotype origin of markers of dosage 1 from a duplex marker in a tetraploid species. Certain combinations of markers provide very unambiguous information about co-inheritance, whereas others do not. Therefore, weights are required to prevent imprecise combinations from exerting a large influence on the map order. Before MDSMap was developed, the only reliable algorithm for ordering weighted recombination frequencies was the weighted regression algorithm from the original JoinMap implementation (Stam, 1993; Van Ooijen, 2006). However, this has the disadvantage of being very slow for higher numbers of marker and is therefore of limited use with current high-density marker datasets. The MDSMap approach can achieve similar results in a fraction of the time, and takes as its input the same information as JoinMap does, the pairwise recombination frequency estimates and logarithm of odds (LOD) scores, making this tool suitable for linkage map construction at any ploidy level, provided pairwise linkage analysis can be performed.

One final tool that has also proven useful for polyploid linkage map construction is the LPmerge package in R (Endelman and Plomion, 2014). LPmerge uses linear programming to remove the minimum number of constraints in marker order in order to create a conflict-free consensus map. It was originally developed to create integrated genetic maps from multiple (diploid) populations. That said, polyploids contain multiple copies of each chromosome and therefore also present a similar challenge if we consider each homolog map as originating from a different population, with non-simplex markers as bridging markers (mapped in more than one population). Homolog-specific maps are still regularly generated in polyploid mapping studies [e.g., in potato (Bourke et al., 2015, 2016), rose (Vukosavljev et al., 2016) or sweet potato (Shirasawa et al., 2017)], for which LPmerge (or a similarly efficient integration algorithm) could then be used to generate chromosomally integrated maps.

### Physical Maps

Arguably, one of the most important “tools” in current genomics studies is access to a high-quality reference genome assembly. Species for which a reference genome assembly exists have even been classified as “model organisms” (Seeb et al., 2011), such is the importance and impact a genome can bring to research on that species. Without a reference sequence available, the scope of genomic research remains limited. For example, GWAS rely on knowledge of the relative position of SNP markers (usually on a physical map), and many sequencing applications rely on a reference assembly on which to map reads. A reference genome also facilitates the development of molecular markers (e.g., primer development), the comparison of results between different genetic studies (by providing a single reference map), as well as allowing comparisons of specific sequences such as genes, enabling prediction of gene function across related species.

Polyploid genomes are by definition more complex than diploid genomes, having multiple copies of each homologous chromosome. Many polyploid species are also outbreeding, leading to increased heterozygosity which is problematic in *de novo* assemblies and necessitates specialized approaches (Kajitani et al., 2014). The most common solution until now has been to sequence a representative diploid species. For example in highly heterozygous autotetraploid potato, a completely homozygous doubled monoploid (*S. tuberosum* group *Phureja* DM1-3) was sequenced (Potato Genome Sequencing Consortium, 2011) which still represents the primary reference sequence today^3^. In the case of allopolyploids, multiple diploid progenitor species are often sequenced instead [e.g., peanut (Bertioli et al., 2016)]. The emergence of the pan-genome concept, originally proposed for microbial species (Tettelin et al., 2005), has interesting implications for how highly heterozygous polyploid genomes will be presented in future. We have already mentioned the arrival of phased genomics with the sweet potato genome, which aimed to generate six chromosome-length phased assemblies for each of its 15 chromosomes (Yang et al., 2017a). In future, both pan-genomes and phased genomes are likely to play a bigger role in polyploid reference genomics. Examples of polyploid species that have so far been “sequenced” are listed in **Table 1**. This is by no means an exhaustive list, nor does it describe all developments for the listed species. For example, the sequence of allotetraploid *Coffea arabica* (which accounts for roughly 70% of all coffee production) has recently been assembled, with a draft assembly (*C. arabica* UCDv0.5) available on the Phytozome database^4^. What **Table 1** highlights is that at the time of writing, there were already a wide range of polyploid crop species that have well-developed genomic resources, despite the fact that in many cases these are from closely related or progenitor diploid species. In time, just like for coffee, we predict that direct sequencing of polyploid species themselves will gradually replace the haploidised reference sequences in importance and application, leading to more insights of direct relevance to polyploids.

**Table 1:** Some examples of publicly available reference sequences for polyploid species.

Target species	Sequenced species (ploidy)	Genome browser	Reference
**Autopolyploids**			
Alfalfa, *Medicago sativa* (4×)	*Medicago truncatula* (2×)	medicagogenome.org | plants.ensembl.org	Young et al., 2011; Tang et al., 2014
Kiwifruit, *Actinidia chinensis* (6×)	*Actinidia chinensis* (2×)	bdg.hfut.edu.cn/kir | bioinfo.bti.cornell.edu/cgi-bin/kiwi/home.cgi	Huang et al., 2013
Potato, *Solanum tuberosum* (4×)	*Solanum tuberosum* (2×)	solanaceae.plantbiology.msu.edu |plants.ensembl.org	Potato Genome Sequencing Consortium, 2011
Sweet potato, *Ipomoea batatas* (6×)	*Ipomoea batatas* (6×)	public-genomes-ngs.molgen.mpg.de/SweetPotato |ipomoea-genome.org	Yang et al., 2017a
Rose, *Rosa* × *hybrida* (4×)	*Rosa chinensis* (2×)	https: //iris.angers.inra.fr/obh/	Hibrand-Saint Oyant et al., unpublished
**Allopolyploids**			
Banana, *Musa acuminata* (3×)	*Musa acuminata* (2×)	banana-genome-hub.southgreen.fr |plants.ensembl.org	D’Hont et al., 2012
Coffee, *Coffea arabica* (4×)	*Coffea canephora* (2×)	coffee-genome.org	Denoeud et al., 2014
Cotton, *Gossypium hirsutum* (4×)	*Gossypium hirsutum* (4×)	cottongen.org	Li et al., 2015
Oilseed rape, *Brassica napus* (4×)	*Brassica napus* (4×)	genoscope.cns.fr/brassicanapus | plants.ensembl.org	Chalhoub et al., 2014
Peanut, *Arachis hypogaea* (4×)	*Arachis duranensis* (2×) *Arachis ipaensis* (2×)	peanutbase.org	Bertioli et al., 2016
Quinoa, *Chenopodium quinoa* (4×)	*Chenopodium quinoa* (4×)	cbrc.kaust.edu.sa/chenopodiumdb	Jarvis et al., 2017
Strawberry, *Fragaria* × *ananassa* (8×)	*Fragaria vesca* (2×)	rosaceae.org	Shulaev et al., 2011
Wheat, *Triticum aestivum* (6×)	*Triticum aestivum* (6×)	wheat-urgi.versailles.inra.fr | plants.ensembl.org	International Wheat Genome Sequencing Consortium, 2014

## Quantitative Trait Analysis and Genomic Selection

One of the main goals of genetic studies is to find causative associations between DNA polymorphisms and phenotypic traits. In domesticated species in particular, these studies are often performed with a practical aim: to develop marker-based methods of selecting superior lines in a breeding program. Traditional approaches such as bi-parental QTL mapping have been complemented in recent years by new methodologies such as GWAS and genomic selection. However, all these approaches require polyploid-specific solutions which can capture the increased complexity of polysomic inheritance. We look at the three most commonly used approaches for identifying quantitative trait variation and how specific software tools are helping to revolutionize polyploid plant breeding programs.

### QTL Analysis

The term “QTL analysis” usually refers to studies that aim to detect regions of the genome [so-called quantitative trait loci (Geldermann, 1975)] that have a significant statistical association with a trait in specifically constructed experimental populations. These populations are most often created by crossing two contrasting parental lines (“bi-parental” populations), although there is increasing interest in using more complex population designs in order to increase the range of alleles and genetic backgrounds being studied [e.g., “MAGIC” populations (Huang et al., 2015)]. As already discussed, there is great difficulty in developing inbred lines by repeatedly selfing polyploids due to the sampling of alleles during polyploid gamete formation [in a diploid this sampling generates $\left(\begin{array}{l} 2\\ 1 \end{array}\right)=2$ combinations; for a tetraploid this rises to $\left(\begin{array}{l} 4\\ 2 \end{array}\right)=6$ and in a hexaploid $\left(\begin{array}{l} 6\\ 3 \end{array}\right)=20$ combinations, resulting in protracted heterozygosity (**Figure 4**)], not to mention the problem of inbreeding depression associated with many outcrossing polyploid species. Therefore, most QTL analyses in polyploid species have been performed using the directly segregating F_1_ progeny of a cross between heterozygous parents (a “full sib” population). This leads to poor resolution of QTL positions when compared to the more popular diploid inbred populations like RILs etc., as well as the fact that populations must be vegetatively propagated if replication over years or different growing environments is desired. For many polyploid species, vegetative propagation is indeed possible (Herben et al., 2017) and F_1_ populations have the added advantage of being relatively quick and simple to develop, while, because of a generally high level of heterozygosity, many loci will be segregating in the F_1_. Therefore despite their drawbacks, F_1_ populations remain the bi-parental population of choice for mapping studies.

The methods for QTL analysis in diploid species have become increasingly convoluted (van Eeuwijk et al., 2010); in polyploid species such theoretical complexities have yet to be attempted, given the more immediate difficulties in accurately genotyping as well as modeling polyploid inheritance. Just like for linkage mapping and GWAS, the range of software tools available for QTL analysis in polyploids remains rather limited, although there are a number of recent developments that are helping transform the field.

One of the only dedicated software for tetraploid QTL analysis is the already-mentioned TetraploidMap software (Hackett et al., 2007). This software enables interval mapping to be performed in autotetraploid F_1_ populations (as well as a simple single-marker ANOVA test), using a restricted range of markers (1 × 0, 2 × 0, and 1 × 1 markers only, where 1 × 0 denotes a marker dosage of 1 in one parent and 0 in the other, etc.). Although still available, it has been superseded by the TetraploidSNPMap software (Hackett et al., 2017). TetraploidSNPMap (TSNPM) uses SNP dosage data to either construct a linkage map (as already described) or perform QTL interval mapping. In contrast to its predecessor, TSNPM can analyze all marker segregation types, and allows the user to explore different QTL models at detected peaks. At its core is an algorithm to determine identity-by-descent (IBD) probabilities for the offspring of the population, which are then used in a weighted regression performed across the genome.

An independent software tool that has been developed to determine IBD probabilities in tetraploids is TetraOrigin (Zheng et al., 2016), implemented in the Mathematica programming language. TetraOrigin relaxes the assumption of random bivalent pairing during meiosis (which TSNPM employs) to allow for both preferential chromosomal pairing as well as multivalent formation and the possibility of double reduction. Although not programmed in a user-friendly format like TSNPM, it is relatively straightforward to use, taking an integrated linkage map and marker dosage matrix as input. It does not perform QTL analysis directly, but the resulting IBD probabilities can then be used to model genotype effects in a QTL scan either using a weighted regression approach like TSNPM, or in a linear mixed model setting. IBD probabilities allow interval mapping since they can be interpolated at any desired intervals on the linkage map.

For ploidy levels other than tetraploid, there are currently no dedicated software tools available for QTL analysis or IBD probability estimation. Single-marker approaches such as ANOVA on the marker dosages [assuming additivity – various dominant models could also be explored; see, e.g., (Rosyara et al., 2016)] are of course possible and require access to basic statistical software packages such as R (or even Excel). However, such approaches are not ideal – they are only effective if marker alleles are closely linked in coupling with QTL alleles, and offer no ability to predict the QTL segregation type or mode of gene action as is done for example in TSNPM (Hackett et al., 2017). As interest increases in the genetic dissection of important traits in polyploid species, we anticipate that it is only a matter of time before more flexible cross-ploidy solutions are developed. Methodologies developed for tetraploid species often claim that “extension to higher ploidy levels is straightforward.” These sorts of disingenuous claims attempt to mark new research territory as already solved. If extensions to higher ploidy levels were indeed straightforward we would already be reporting on a wider range of tools available for them – as far as we can tell, so far there are none.

Returning to the topic of population types, we also anticipate that more powerful QTL analyses can be performed by combining information over multiple populations. Approaches such as pedigree-informed analyses, implemented for diploids in the FlexQTL software (Bink et al., 2008), could overcome some of the limitations imposed by the restrictions on population types in software for polyploids. However, it may take some time before such tools become translated to the polyploid level.

### Genome-Wide Association Studies

Genome-wide association studies have emerged as a powerful tool for detecting causative loci underlying phenotypic traits. They have been particularly popular in species where the generation of experimental populations is problematic (such as humans). GWAS has been readily adopted across a broad spectrum of species since then, due to the promise of increased mapping resolution, a more diverse sampling of alleles and a simplicity in population creation (no crossing required) (Bernardo, 2016). There are certain disadvantages though, particularly in how rare (and potentially important) variants can be missed (Ott et al., 2015) and the confounding effect of population structure on results (Korte and Farlow, 2013). Nevertheless, GWAS continues to be an important analytical option to help shed greater light on genotype – phenotype associations. The application of GWAS in polyploid species is relatively new, although there have already been a number of studies published in various crop species, for example in potato, oilseed rape, wheat, and oats (Uitdewilligen et al., 2013; Gajardo et al., 2015; Sukumaran et al., 2015; Tumino et al., 2016, 2017). GWAS studies usually need to account for population structure and relatedness to prevent spurious associations, often in the context of linear mixed models (Yu et al., 2006; Bradbury et al., 2007; Zhang et al., 2010).

One challenge in applying GWAS to polyploid species is how to define a relatedness metric between polyploid individuals (i.e., how to generate the kinship matrix, *K*). So far, there have been two software tools released for polyploid GWAS, namely the R package GWASpoly (Rosyara et al., 2016) and the previously mentioned SHEsisPlus (Shen et al., 2016). Of these, only GWASpoly looks critically at the form of the kinship matrix *K*. Three different forms of *K* were tested in the development of the package, with the canonical relationship matrix (VanRaden, 2008) [termed the realized relationship matrix by the authors (Rosyara et al., 2016)] found to best control against inflation of significance values. This is also the default *K* provided in the GWASpoly package. An alternative approach to GWAS mapping for polyploids is provided by the netgwas package (Behrouzi and Wit, 2017b), previously mentioned for its linkage mapping capacity. Again, graphical models form the basis of the approach, which goes beyond single-marker association mapping to investigate genotype-phenotype interactions using all markers simultaneously in a graph structure. There is almost no discussion on how confoundedness between population structure and phenotypes are handled, but the authors claim the detection of false positive associations is not problematic.

One final aspect worth considering is the issue of deploying an adequate number of markers in a polyploid GWAS, which potentially represents a much larger genomic space. In *A. thaliana*, it was estimated that between 140K and 250K SNPs would be needed to fully cover the genome based on a study of linkage disequilibrium in that species (Kim et al., 2007). Modeling the decay of linkage disequilibrium in polyploid species is a more complex exercise. It was previously suggested that estimates of linkage disequilibrium may be inflated in polyploid species (Jannoo et al., 1999; Flint-Garcia et al., 2003). A more recent survey of linkage disequilibrium in autotetraploid potato using SNP dosages estimated that at most 40K SNPs would be needed for QTL discovery in potato (Vos et al., 2017), a much lower estimate than for *Arabidopsis* (Kim et al., 2007). The discrepancy comes in part from the differences in how these figures were estimated, using a ‘hide-the-SNP’ simulation for *Arabidopsis* versus a ‘rule of thumb’ calculation for potato, but mainly from the difference in the extent of LD between the two species [estimated at ∼10 Kb in *A. thaliana* versus ∼2 Mb in *S. tuberosum* (Kim et al., 2007; Vos et al., 2017)]. Detecting or even defining linkage disequilibrium between markers linked in repulsion phase is non-trivial in autopolyploids (Vos et al., 2017), which is analogous to the problem of detecting and estimating recombination frequency between such markers in a linkage mapping study. So far, we are not aware of any software tool that has been developed to estimate the extent of linkage disequilibrium in polyploids, which would complement the design of future GWAS studies in polyploid species.

### Genomic Prediction and Genomic Selection

There has been much attention given to the advantages of using *all* marker data to help predict phenotypic performance, rather than focussing on single markers (or haplotypes) that are linked to QTL as was previously advocated. The motivation behind this is clear – many of the most important traits in domesticated animal and plant species are highly quantitative, with far too many small-effect loci present to be able to tag them all with single markers (Bernardo, 2008). One of the most important traits in any breeding program is also a famously quantitative trait: yield. It has been suggested that despite many years of phenotypic selection, crop yield in tetraploid potato has essentially remained unchanged (Jansky, 2009; Slater et al., 2016). This is a remarkable indictment of traditional selection methods, yet offers much-needed impetus for the development and deployment of new paradigms in breeding for quantitative traits.

Genomic prediction first arose in animal breeding circles (Meuwissen et al., 2001), where the concept of estimating breeding values from known pedigrees was already well-established. However, the estimation of breeding values in polyploid species requires special consideration due to the complexity of polysomic inheritance and the possibility of double reduction. In practice, breeding values are usually estimated using restricted maximum likelihood (REML) to solve mixed model equations, requiring the generation of an inverse additive relationship matrix *A^-1^*, also called the numerator relationship matrix. The form of *A^-1^* depends on, among other things, whether the inheritance is polysomic or disomic, and whether double reduction occurs (Kerr et al., 2012; Amadeu et al., 2016; Hamilton and Kerr, 2017). The R package AGHmatrix was developed in order to compute the appropriate *A* matrix for autotetraploids with a known pedigree (Amadeu et al., 2016), using theory developed in (Kerr et al., 2012). In applying their approach to an autotetraploid blueberry (*Vaccinium corymbosum* L.) population, the authors determined the *A* matrix under various levels of double reduction, afterwards selecting the model which maximized the likelihood of the data (Amadeu et al., 2016). More recently, an alternative R package polyAinv was released which computes *A^-1^* as well as the kinship matrix *K* and the inbreeding coefficients *F* (Hamilton and Kerr, 2017). polyAinv claims to be applicable to any ploidy level (rather than just autotetraploids) and can accommodate sex-based differences in IBD probabilities (Hamilton and Kerr, 2017). Like AGHmatrix, it also incorporates double reduction in its calculations. However, in one study of nine common traits in autotetraploid potato, the inclusion of double reduction, or even the adoption of an autotetraploid-appropriate relationship matrix was found to have a minimal impact on the results (Slater et al., 2014). Studies which ignore the specific complexities of autopolyploids may still benefit from genomic prediction and selection, as for example was demonstrated in tetraploid potato (Sverrisdóttir et al., 2017). Commonly used software tools for estimating breeding values at the diploid level include ProGeno (Maenhout, 2018) and ASreml (VSN International, 2018) which could be suitable for polyploid breeding programs, although this has yet to be conclusively demonstrated.

## Polyploid Inheritance and Simulation

As a final section we look at two topics which are important to the development of polyploid genetic resources – the mode of inheritance and the availability of simulation software for polyploid species. Although these topics do not necessarily go together, they represent very important considerations in themselves. The mode of inheritance is a polyploid-specific topic, with no equivalent issue arising in diploid genetic studies. Simulation studies, on the other hand, have been used repeatedly at the diploid level to test new methodologies, determine empirical thresholds, evaluate competing methods etc. The availability of a range of software options to simulate polyploid genetic behavior is crucial if polyploid genetics is to flourish.

### Mode of Inheritance

The term “mode of inheritance” refers to the randomness of meiotic pairing processes that give rise to gametes, and is often used to distinguish between disomic (diploid-like) inheritance, and polysomic (all allele combinations equally possible) inheritance. As alluded to already, intermediate modes of inheritance are theoretically possible if partially preferential pairing occurs between homologs, resulting in on average more recombinations between certain homologs, and less between others (putative homoeologs). This intermediate inheritance pattern, originally termed segmental allopolyploidy (Stebbins, 1947) and more recently termed mixosomy (Soltis et al., 2016), poses additional challenges over those of purely polysomic or disomic behavior. One of the main complications is the lack of fixed segregation ratios to test markers against (Allendorf and Danzmann, 1997), which is often used as a measure of marker quality (Stringham and Boehnke, 1996; Pompanon et al., 2005). Currently there are no dedicated tools available to ascertain the most likely mode of inheritance in polyploids. Some “traditional” approaches to predict the mode of inheritance are summarized in (Bourke et al., 2017), many of which are relatively straightforward to implement using a statistical programming environment like R (R Core Team, 2016). In that study, TetraOrigin (Zheng et al., 2016) was used to estimate the most likely pairing configuration that gave rise to each offspring in an F_1_ tetraploid population. This enabled the authors to test whether there were deviations from the expected patterns of homolog pairing under a tetrasomic model (Bourke et al., 2017). A simple alternative using closely linked repulsion-phase simplex marker pairs was also proposed and has been implemented in the polymapR package (Bourke et al., unpublished). Apart from preferential pairing, TetraOrigin can also predict whether marker data arose from bivalent or multivalent pairing during meiosis, facilitating an analysis of the distribution of double reduction products. However, apart from its restriction to tetraploid data, an integrated linkage map is required before TetraOrigin can be employed. In severe cases of mixosomy, it is not obvious how a reliable linkage map should be generated. Corrections for mixosomy in a tetraploid linkage analysis are possible in polymapR, but in extreme cases marker clustering will also be affected, making map construction quite challenging. A confounding complication is the possibility of variable chromosome counts (aneuploidy), as for example encountered in sugarcane (Grivet et al., 1996; Grivet and Arruda, 2002) or in ornamentals such as *Alstroemeria* (Buitendijk et al., 1997), which makes the diagnosis of the mode of inheritance even more difficult. As more polyploid species begin to be genotyped, the issue of unknown mode of inheritance will likely exert more influence, further necessitating the development of software tools that can provide an accurate assessment of the inheritance mode using marker data, and that can accommodate the full spectrum of polyploid meiotic behaviors.

### Simulation Software

As with any software tool, developing standards and scenarios upon which the performance of the tool can be judged is vital to ensure reliable results. In this final section we consider the range of simulation tools currently available for polyploids. Probably the most widely used polyploid simulation software currently available is PedigreeSim (Voorrips and Maliepaard, 2012). Originally developed to generate diploid and tetraploid populations, the current release (PedigreeSim V2.0) can simulate populations of any even ploidy level (2, 4, 6, …). What makes PedigreeSim particularly attractive is its ability to simulate a diversity of meiotic pairing conditions, including quadrivalents (which can result in double reduction) or preferential chromosome pairing. It takes four input files (which are relatively simple to generate) that provide a description of the desired simulation parameters and the input marker data. The software then creates (dosage-scored) genotype data for any pedigreed population, e.g., an F_1_ population of specified size (Voorrips and Maliepaard, 2012). Some authors have used PedigreeSim to simulate multiple generations of random mating, allowing an investigation of population structure and linkage disequilibrium in polyploid species (e.g., Rosyara et al., 2016; Vos et al., 2017), which can be implemented quite easily with some basic programming knowledge. PedigreeSim is written in Java and can run on all major operating systems.

A Windows-based software Polylink, which originally performed two-point linkage analysis and simulation of tetraploid populations (He et al., 2001), is no longer available. The R package polySegratio (Baker, 2014) simulates dominantly scored marker data in autopolyploids of any even ploidy level. Generating the dosage data is straightforward: only the expected proportion of marker types (simplex, duplex, triplex, …) as well as the ploidy is required. However, the markers are essentially completely random, with no connection to any linkage map, which is arguably of limited use for any application that requires some degree of linkage between markers. The simulation capacities of polysegRatio therefore appear to be most useful for testing functions within the package itself, namely those designed to impute parental dosages given the observed segregation ratios in offspring scores.

A final polyploid simulation tool that has recently been developed is the HaploSim pipeline which includes the HaploGenerator function (Motazedi et al., 2017). HaploGenerator is designed to generate sequence-based haplotypes in a polyploid of any even ploidy, taking the fasta file it is provided with as a reference from which haplotypes are built. The software generates random SNP mutations at a specified distribution before simulating next-generation sequencing (NGS) reads in formats corresponding to a number of current sequencing technologies such as Illumina or Pacific Biosystems (PacBio). The pipeline was originally developed to compare the performance of a number of haplotype assembly algorithms (Motazedi et al., 2017), but could also be useful for testing the performance of any other tool which uses NGS reads as genotypes.

## Future Perspectives

In this review we have attempted to describe the most important software tools that are currently available to the polyploid genetics community. There are likely to be tools that were missed and tools that have subsequently been released – this is the danger of such a review. However, we have tried where possible to also discuss the gaps that are apparent in the current set of available tools which will hopefully help guide their development in future. Polyploid genotyping arguably remains the most critical step, as without accurate genotype data there is little point in building models for polyploid inheritance. However, we are now witnessing the slow emergence of tools that take polyploid genotypes and use them to make inferences on the transmission of alleles and the effects of such alleles in polyploid populations. As genotyping technologies continue to evolve, so too should the suite of tools developed to analyze those genotypes. Tools for analyzing SNP dosage data from SNP arrays are well-established. The coming decade will likely see a move away from SNP array-based genotyping to the use of sequence-read based genotypes, although this will require that all tools heretofore developed be updated to accommodate the new type of data. Information on the mode of inheritance from marker data is also needed for each population studied, which deserves more attention than it currently receives. A move from diploid-based reference genomes to fully polyploid (and haplotype-resolved) reference genomes would also help broaden the boundaries of polyploid genetics away from the diplo-centric view of genomics which currently dominates. Although there have been many exciting discoveries and developments in polyploid genetics in the past decade or more, we feel its golden age has yet to arrive, an age which will be heralded all the sooner by the provision of robust and user-friendly tools for the genetic dissection of this fascinating group of organisms.

## Author Contributions

PB wrote this review, with input from REV, RGFV, and CM. All authors read and approved the final manuscript.

## Conflict of Interest Statement

The authors of this review have been involved in the development of a number of the software tools mentioned, namely fitTetra, fitPoly, PedigreeSim, TetraOrigin, polymapR, HaploSim, and TriPoly. We have tried to give an impartial perspective where these and alternative tools are concerned. Apart from this, the authors do not have any further conflicts of interest to declare.

## Footnotes

1 https://cran.r-project.org/package=fitPoly

2 https://bioss.ac.uk/knowledge/tetramap.html

3 http://solanaceae.plantbiology.msu.edu/

4 www.phytozome.net

## References

[B1] Aguiar D. Istrail S. (2013). Haplotype assembly in polyploid genomes and identical by descent shared tracts. *Bioinformatics* 29 i352–i360. 10.1093/bioinformatics/btt213 23813004 PMC3694639

[B2] Akhunov E. Nicolet C. Dvorak J. (2009). Single nucleotide polymorphism genotyping in polyploid wheat with the Illumina GoldenGate assay. *Theor. Appl. Genet.* 119 507–517. 10.1007/s00122-009-1059-5 19449174 PMC2715469

[B3] Albrechtsen A. Nielsen F. C. Nielsen R. (2010). Ascertainment biases in SNP chips affect measures of population divergence. *Mol. Biol. Evol.* 27 2534–2547. 10.1093/molbev/msq148 20558595 PMC3107607

[B4] Allendorf F. W. Danzmann R. G. (1997). Secondary tetrasomic segregation of MDH-B and preferential pairing of homeologues in rainbow trout. *Genetics* 145 1083–1092. 9093860 10.1093/genetics/145.4.1083 PMC1207878

[B5] Amadeu R. R. Cellon C. Olmstead J. W. Garcia A. A. Resende M. F. Muñoz P. R. (2016). AGHmatrix: R Package to construct relationship matrices for autotetraploid and diploid species: a blueberry example. *Plant Genome* 9 1–10. 10.3835/plantgenome2016.01.0009 27902800

[B6] Bajgain P. Rouse M. N. Anderson J. A. (2016). Comparing genotyping-by-sequencing and single nucleotide polymorphism chip genotyping for quantitative trait loci mapping in wheat. *Crop Sci.* 56 232–248. 10.2135/cropsci2015.06.0389

[B7] Baker P. (2014). polySegratio: simulate and test marker dosage for dominant markers in autopolyploids. R Package Version 0.2–4. 10.1007/s00122-010-1283-z 20182697

[B8] Baker P. Jackson P. Aitken K. (2010). Bayesian estimation of marker dosage in sugarcane and other autopolyploids. *Theor. Appl. Genet.* 120 1653–1672. 10.1007/s00122-010-1283-z 20182697

[B9] Balsalobre T. W. A. Da Silva Pereira G. Margarido G. R. A. Gazaffi R. Barreto F. Z. Anoni C. O. (2017). GBS-based single dosage markers for linkage and QTL mapping allow gene mining for yield-related traits in sugarcane. *BMC Genomics* 18:72. 10.1186/s12864-016-3383-x 28077090 PMC5225503

[B10] Barker M. S. Arrigo N. Baniaga A. E. Li Z. Levin D. A. (2016). On the relative abundance of autopolyploids and allopolyploids. *New Phytol.* 210 391–398. 10.1111/nph.13698 26439879

[B11] Barringer B. C. (2007). Polyploidy and self-fertilization in flowering plants. *Am. J. Bot.* 94 1527–1533. 10.3732/ajb.94.9.1527 21636519

[B12] Bartholomé J. Mandrou E. Mabiala A. Jenkins J. Nabihoudine I. Klopp C. (2015). High-resolution genetic maps of *Eucalyptus* improve *Eucalyptus grandis* genome assembly. *New Phytol.* 206 1283–1296. 10.1111/nph.13150 25385325

[B13] Bassil N. V. Davis T. M. Zhang H. Ficklin S. Mittmann M. Webster T. (2015). Development and preliminary evaluation of a 90 K Axiom^®^ SNP array for the allo-octoploid cultivated strawberry *Fragaria × ananassa*. *BMC Genomics* 16:155. 10.1186/s12864-015-1310-1 25886969 PMC4374422

[B14] Behrouzi P. Wit E. C. (2017a). De novo construction of q-ploid linkage maps using discrete graphical models. arXiv preprint arXiv:1710.01063. 10.1093/bioinformatics/bty777 30184062

[B15] Behrouzi P. Wit E. C. (2017b). netgwas: an R package for network-based genome-wide association studies. arXiv preprint arXiv:1710.01236.

[B16] Berger E. Yorukoglu D. Peng J. Berger B. (2014). Haptree: A novel Bayesian framework for single individual polyplotyping using NGS data. *PLoS Comput. Biol.* 10:e1003502. 10.1371/journal.pcbi.1003502 24675685 PMC3967924

[B17] Bernardo R. (2008). Molecular markers and selection for complex traits in plants: learning from the last 20 years. *Crop Sci.* 48 1649–1664. 10.2135/cropsci2008.03.0131

[B18] Bernardo R. (2016). Bandwagons I, too, have known. *Theor. Appl. Genet.* 129 2323–2332. 10.1007/s00122-016-2772-5 27681088

[B19] Bertioli D. J. Cannon S. B. Froenicke L. Huang G. Farmer A. D. Cannon E. K. (2016). The genome sequences of *Arachis duranensis* and *Arachis ipaensis*, the diploid ancestors of cultivated peanut. *Nat. Genet.* 48 438–446. 10.1038/ng.3517 26901068

[B20] Bink M. Boer M. Ter Braak C. Jansen J. Voorrips R. Van De Weg W. (2008). Bayesian analysis of complex traits in pedigreed plant populations. *Euphytica* 161 85–96. 10.1007/s10681-007-9516-1

[B21] Bourke P. M. Arens P. Voorrips R. E. Esselink G. D. Koning-Boucoiran C. F. S. Van ‘t Westende W. P. C. (2017). Partial preferential chromosome pairing is genotype dependent in tetraploid rose. *Plant J.* 90 330–343. 10.1111/tpj.13496 28142191

[B22] Bourke P. M. Voorrips R. E. Kranenburg T. Jansen J. Visser R. G. Maliepaard C. (2016). Integrating haplotype-specific linkage maps in tetraploid species using SNP markers. *Theor. Appl. Genet.* 129 2211–2226. 10.1007/s00122-016-2768-1 27561740 PMC5069339

[B23] Bourke P. M. Voorrips R. E. Visser R. G. F. Maliepaard C. (2015). The double reduction landscape in tetraploid potato as revealed by a high-density linkage map. *Genetics* 201 853–863. 10.1534/genetics.115.181008 26377683 PMC4649655

[B24] Bradbury P. J. Zhang Z. Kroon D. E. Casstevens T. M. Ramdoss Y. Buckler E. S. (2007). TASSEL: software for association mapping of complex traits in diverse samples. *Bioinformatics* 23 2633–2635. 10.1093/bioinformatics/btm308 17586829

[B25] Buitendijk J. H. Boon E. J. Ramanna M. S. (1997). Nuclear DNA content in twelve species of *Alstroemeria* L. and some of their hybrids. *Ann. Bot.* 79 343–353. 10.1006/anbo.1996.0345 28403819

[B26] Bushman B. Robbins M. Larson S. Staub J. (eds) (2016). “Genotyping by sequencing in autotetraploid cocksfoot (*Dactylis glomerata*) without a reference genome,” in *Breeding in a World of Scarcity* (Berlin: Springer) 133–137. 10.1007/978-3-319-28932-8_20

[B27] Cavanagh C. R. Chao S. Wang S. Huang B. E. Stephen S. Kiani S. (2013). Genome-wide comparative diversity uncovers multiple targets of selection for improvement in hexaploid wheat landraces and cultivars. *Proc. Natl. Acad. Sci. U.S.A.* 110 8057–8062. 10.1073/pnas.1217133110 23630259 PMC3657823

[B28] Chaffin A. S. Huang Y.-F. Smith S. Bekele W. A. Babiker E. Gnanesh B. N. (2016). A consensus map in cultivated hexaploid oat reveals conserved grass synteny with substantial subgenome rearrangement. *Plant Genome* 9 1–21. 10.3835/plantgenome2015.10.0102 27898818

[B29] Chalhoub B. Denoeud F. Liu S. Parkin I. A. Tang H. Wang X. (2014). Early allopolyploid evolution in the post-Neolithic *Brassica napus* oilseed genome. *Science* 345 950–953. 10.1126/science.1253435 25146293

[B30] Cheema J. Dicks J. (2009). Computational approaches and software tools for genetic linkage map estimation in plants. *Brief. Bioinform.* 10 595–608. 10.1093/bib/bbp045 19933208

[B31] Chester M. Gallagher J. P. Symonds V. V. Da Silva A. V. C. Mavrodiev E. V. Leitch A. R. (2012). Extensive chromosomal variation in a recently formed natural allopolyploid species, *Tragopogon miscellus* (Asteraceae). *Proc. Natl. Acad. Sci. U.S.A.* 109 1176–1181. 10.1073/pnas.1112041109 22228301 PMC3268322

[B32] Clarke W. E. Higgins E. E. Plieske J. Wieseke R. Sidebottom C. Khedikar Y. (2016). A high-density SNP genotyping array for *Brassica napus* and its ancestral diploid species based on optimised selection of single-locus markers in the allotetraploid genome. *Theor. Appl. Genet.* 129 1887–1899. 10.1007/s00122-016-2746-7 27364915 PMC5025514

[B33] Comai L. (2005). The advantages and disadvantages of being polyploid. *Nat. Rev. Genet.* 6 836–846. 10.1038/nrg1711 16304599

[B34] Cornille A. Salcedo A. Kryvokhyzha D. Glémin S. Holm K. Wright S. I. (2016). Genomic signature of successful colonization of Eurasia by the allopolyploid shepherd’s purse (*Capsella bursa-pastoris*). *Mol. Ecol.* 25 616–629. 10.1111/mec.13491 26607306

[B35] Crawford J. Brown P. J. Voigt T. Lee D. (2016). Linkage mapping in prairie cordgrass (*Spartina pectinata* Link) using genotyping-by-sequencing. *Mol. Breed.* 36:62 10.1007/s11032-016-0484-9

[B36] Dalton-Morgan J. Hayward A. Alamery S. Tollenaere R. Mason A. S. Campbell E. (2014). A high-throughput SNP array in the amphidiploid species *Brassica napus* shows diversity in resistance genes. *Funct. Integr. Genomics* 14 643–655. 10.1007/s10142-014-0391-2 25147024

[B37] Das S. Vikalo H. (2015). SDhaP: haplotype assembly for diploids and polyploids via semi-definite programming. *BMC Genomics* 16:260. 10.1186/s12864-015-1408-5 25885901 PMC4422552

[B38] de Bakker P. I. Yelensky R. Pe’er I. Gabriel S. B. Daly M. J. Altshuler D. (2005). Efficiency and power in genetic association studies. *Nat. Genet.* 37 1217–1223. 10.1038/ng1669 16244653

[B39] Denoeud F. Carretero-Paulet L. Dereeper A. Droc G. Guyot R. Pietrella M. (2014). The coffee genome provides insight into the convergent evolution of caffeine biosynthesis. *Science* 345 1181–1184. 10.1126/science.1255274 25190796

[B40] D’Hont A. Denoeud F. Aury J.-M. Baurens F.-C. Carreel F. Garsmeur O. (2012). The banana (*Musa acuminata*) genome and the evolution of monocotyledonous plants. *Nature* 488 213–217. 10.1038/nature11241 22801500

[B41] Didion J. P. Yang H. Sheppard K. Fu C.-P. Mcmillan L. De Villena F. P.-M. (2012). Discovery of novel variants in genotyping arrays improves genotype retention and reduces ascertainment bias. *BMC Genomics* 13:34. 10.1186/1471-2164-13-34 22260749 PMC3305361

[B42] Doyle J. J. Sherman-Broyles S. (2016). Double trouble: taxonomy and definitions of polyploidy. *New Phytol.* 213 487–493. 10.1111/nph.14276 28000935

[B43] Edae E. A. Bowden R. L. Poland J. (2015). Application of population sequencing (POPSEQ) for ordering and imputing genotyping-by-sequencing markers in hexaploid wheat. *G*3 5 2547–2553. 10.1534/g3.115.020362 26530417 PMC4683627

[B44] Endelman J. B. Plomion C. (2014). LPmerge: an R package for merging genetic maps by linear programming. *Bioinformatics* 30 1623–1624. 10.1093/bioinformatics/btu091 24532720

[B45] Felcher K. J. Coombs J. J. Massa A. N. Hansey C. N. Hamilton J. P. Veilleux R. E. (2012). Integration of two diploid potato linkage maps with the potato genome sequence. *PLoS One* 7:e36347. 10.1371/journal.pone.0036347 22558443 PMC3338666

[B46] Fierst J. L. (2015). Using linkage maps to correct and scaffold de novo genome assemblies: methods, challenges, and computational tools. *Front. Genet.* 6:220. 10.3389/fgene.2015.00220 26150829 PMC4473057

[B47] Flint-Garcia S. A. Thornsberry J. M. Buckler E. S. IV. (2003). Structure of linkage disequilibrium in plants. *Annu. Rev. Plant Biol.* 54 357–374. 10.1146/annurev.arplant.54.031902.134907 14502995

[B48] Gajardo H. A. Wittkop B. Soto-Cerda B. Higgins E. E. Parkin I. A. P. Snowdon R. J. (2015). Association mapping of seed quality traits in *Brassica napus* L. using GWAS and candidate QTL approaches. *Mol. Breed.* 35:143 10.1007/s11032-015-0340-3

[B49] Galloway L. F. Etterson J. R. (2007). Inbreeding depression in an autotetraploid herb: a three cohort field study. *New Phytol.* 173 383–392. 10.1111/j.1469-8137.2006.01909.x 17204084

[B50] Galloway L. F. Etterson J. R. Hamrick J. L. (2003). Outcrossing rate and inbreeding depression in the herbaceous autotetraploid, *Campanula americana.* *Heredity* 90 308–315. 10.1038/sj.hdy.6800242 12692584

[B51] Geldermann H. (1975). Investigations on inheritance of quantitative characters in animals by gene markers I. Methods. *TAG Theor. Appl. Genet.* 46 319–330. 10.1007/BF00281673 24420173

[B52] Gidskehaug L. Kent M. Hayes B. J. Lien S. (2010). Genotype calling and mapping of multisite variants using an Atlantic salmon iSelect SNP array. *Bioinformatics* 27 303–310. 10.1093/bioinformatics/btq673 21149341

[B53] Glover N. M. Redestig H. Dessimoz C. (2016). Homoeologs: what are they and how do we infer them? *Trends Plant Sci.* 21 609–621. 10.1016/j.tplants.2016.02.005 27021699 PMC4920642

[B54] Goldschmidt R. (1933). Some aspects of evolution. *Science* 78 539–547. 10.1126/science.78.2033.539 17811930

[B55] Grandke F. Ranganathan S. Van Bers N. De Haan J. R. Metzler D. (2017). PERGOLA: fast and deterministic linkage mapping of polyploids. *BMC Bioinformatics* 18:12. 10.1186/s12859-016-1416-8 28049428 PMC5210299

[B56] Grivet L. Arruda P. (2002). Sugarcane genomics: depicting the complex genome of an important tropical crop. *Curr. Opin. Plant Biol.* 5 122–127. 10.1016/S1369-5266(02)00234-0 11856607

[B57] Grivet L. D’hont A. Roques D. Feldmann P. Lanaud C. Glaszmann J. C. (1996). RFLP mapping in cultivated sugarcane (*Saccharum* spp.): genome organization in a highly polyploid and aneuploid interspecific hybrid. *Genetics* 142 987–1000. 8849904 10.1093/genetics/142.3.987 PMC1207035

[B58] Hackett C. Luo Z. (2003). TetraploidMap: construction of a linkage map in autotetraploid species. *J. Hered.* 94 358–359. 10.1093/jhered/esg066 12920109

[B59] Hackett C. A. Boskamp B. Vogogias A. Preedy K. F. Milne I. (2017). TetraploidSNPMap: Software for linkage analysis and QTL mapping in autotetraploid populations using SNP dosage data. *J. Hered.* 108 438–442. 10.1093/jhered/esx022

[B60] Hackett C. A. Mclean K. Bryan G. J. (2013). Linkage analysis and QTL mapping using SNP dosage data in a tetraploid potato mapping population. *PLoS One* 8:e63939. 10.1371/journal.pone.0063939 23704960 PMC3660524

[B61] Hackett C. A. Milne I. Bradshaw J. E. Luo Z. (2007). TetraploidMap for windows: linkage map construction and QTL mapping in autotetraploid species. *J. Hered.* 98 727–729. 10.1093/jhered/esm086 17965198

[B62] Haldane J. B. (1930). Theoretical genetics of autopolyploids. *J. Genet.* 22 359–372. 10.1007/BF02984197

[B63] Hamilton J. P. Hansey C. N. Whitty B. R. Stoffel K. Massa A. N. Van Deynze A. (2011). Single nucleotide polymorphism discovery in elite north american potato germplasm. *BMC Genomics* 12:302. 10.1186/1471-2164-12-302 21658273 PMC3128068

[B64] Hamilton M. G. Kerr R. J. (2017). Computation of the inverse additive relationship matrix for autopolyploid and multiple-ploidy populations. *Theor. Appl. Genet.* 131 851–860. 10.1007/s00122-017-3041-y 29260268

[B65] Harlan J. R. De Wet J. M. J. (1975). On Ö. Winge and a prayer: the origins of polyploidy. *Bot. Rev.* 41 361–390. 10.1007/BF02860830

[B66] He Y. Xu X. Tobutt K. R. Ridout M. S. (2001). Polylink: to support two-point linkage analysis in autotetraploids. *Bioinformatics* 17 740–741. 10.1093/bioinformatics/17.8.740 11524376

[B67] Herben T. Suda J. Klimešová J. (2017). Polyploid species rely on vegetative reproduction more than diploids: a re-examination of the old hypothesis. *Ann. Bot.* 120 341–349. 10.1093/aob/mcx009 28334206 PMC5737615

[B68] Huang B. E. Verbyla K. L. Verbyla A. P. Raghavan C. Singh V. K. Gaur P. (2015). MAGIC populations in crops: current status and future prospects. *Theor. Appl. Genet.* 128 999–1017. 10.1007/s00122-015-2506-0 25855139

[B69] Huang S. Ding J. Deng D. Tang W. Sun H. Liu D. (2013). Draft genome of the kiwifruit *Actinidia chinensis*. *Nat. Commun.* 4:2640. 10.1038/ncomms3640 24136039 PMC4089393

[B70] Hulse-Kemp A. M. Lemm J. Plieske J. Ashrafi H. Buyyarapu R. Fang D. D. (2015). Development of a 63K SNP Array for cotton and high-density mapping of intra- and inter-specific populations of *Gossypium* spp. *G*3 5 1187–1209. 10.1534/g3.115.018416 25908569 PMC4478548

[B71] International Wheat Genome Sequencing Consortium (2014). A chromosome-based draft sequence of the hexaploid bread wheat (*Triticum aestivum*) genome. *Science* 345:1251788. 10.1126/science.1251788 25035500

[B72] Islam M. S. Thyssen G. N. Jenkins J. N. Fang D. D. (2015). Detection, validation, and application of genotyping-by-sequencing based single nucleotide polymorphisms in Upland cotton. *Plant Genome* 8 1–10. 10.3835/plantgenome2014.07.0034 33228292

[B73] Jannoo N. Grivet L. Dookun A. D’hont A. Glaszmann J. C. (1999). Linkage disequilibrium among modern sugarcane cultivars. *Theor. Appl. Genet.* 99 1053–1060.

[B74] Jansky S. (2009). “Chapter 2 - Breeding, genetics, and cultivar development,” in *Advances in Potato Chemistry and Technology* eds Singh J. Kaur L. (San Diego, CA: Academic Press) 27–62.

[B75] Jarvis D. E. Ho Y. S. Lightfoot D. J. Schmöckel S. M. Li B. Borm T. J. A. (2017). The genome of *Chenopodium quinoa*. *Nature* 542 307–312. 10.1038/nature21370 28178233

[B76] Jones D. B. Jerry D. R. Khatkar M. S. Raadsma H. W. Van Der Steen H. Prochaska J. (2017). A comparative integrated gene-based linkage and locus ordering by linkage disequilibrium map for the Pacific white shrimp, *Litopenaeus vannamei*. *Sci. Rep.* 7:10360. 10.1038/s41598-017-10515-7 28871114 PMC5583237

[B77] Kajitani R. Toshimoto K. Noguchi H. Toyoda A. Ogura Y. Okuno M. (2014). Efficient de novo assembly of highly heterozygous genomes from whole-genome shotgun short reads. *Genome Res.* 24 1384–1395. 10.1101/gr.170720.113 24755901 PMC4120091

[B78] Kantarski T. Larson S. Zhang X. Dehaan L. Borevitz J. Anderson J. (2017). Development of the first consensus genetic map of intermediate wheatgrass (*Thinopyrum intermedium*) using genotyping-by-sequencing. *Theor. Appl. Genet.* 130 137–150. 10.1007/s00122-016-2799-7 27738715

[B79] Kaur S. Francki M. G. Forster J. W. (2012). Identification, characterization and interpretation of single-nucleotide sequence variation in allopolyploid crop species. *Plant Biotechnol. J.* 10 125–138. 10.1111/j.1467-7652.2011.00644.x 21831136

[B80] Kerr R. J. Li L. Tier B. Dutkowski G. W. Mcrae T. A. (2012). Use of the numerator relationship matrix in genetic analysis of autopolyploid species. *Theor. Appl. Genet.* 124 1271–1282. 10.1007/s00122-012-1785-y 22311370

[B81] Kihara H. Ono T. (1926). Chromosomenzahlen und systematische Gruppierung der Rumex-Arten. *Z. Zellforsch. Mikrosk. Anat.* 4 475–481. 10.1007/BF00391215

[B82] Kim C. Guo H. Kong W. Chandnani R. Shuang L.-S. Paterson A. H. (2016). Application of genotyping by sequencing technology to a variety of crop breeding programs. *Plant Sci.* 242 14–22. 10.1016/j.plantsci.2015.04.016 26566821

[B83] Kim S. Plagnol V. Hu T. T. Toomajian C. Clark R. M. Ossowski S. (2007). Recombination and linkage disequilibrium in *Arabidopsis thaliana*. *Nat. Genet.* 39 1151–1155. 10.1038/ng2115 17676040

[B84] Koning-Boucoiran C. F. S. Esselink G. D. Vukosavljev M. Van’t Westende W. P. C. Gitonga V. W. Krens F. A. (2015). Using RNA-Seq to assemble a rose transcriptome with more than 13000 full-length expressed genes and to develop the WagRhSNP 68k Axiom SNP array for rose (Rosa L.). *Front. Plant Sci.* 6:249. 10.3389/fpls.2015.00249 25954285 PMC4404716

[B85] Korte A. Farlow A. (2013). The advantages and limitations of trait analysis with GWAS: a review. *Plant Methods* 9:29. 10.1186/1746-4811-9-29 23876160 PMC3750305

[B86] Krebs S. L. Hancock J. F. (1990). Early-acting inbreeding depression and reproductive success in the highbush blueberry, *Vaccinium corymbosum* L. *Theor. Appl. Genet.* 79 825–832. 10.1007/BF00224252 24226746

[B87] Lachance J. Tishkoff S. A. (2013). SNP ascertainment bias in population genetic analyses: why it is important, and how to correct it. *Bioessays* 35 780–786. 10.1002/bies.201300014 23836388 PMC3849385

[B88] Li F. Fan G. Lu C. Xiao G. Zou C. Kohel R. J. (2015). Genome sequence of cultivated upland cotton (*Gossypium hirsutum* TM-1) provides insights into genome evolution. *Nat. Biotechnol.* 33 524–530. 10.1038/nbt.3208 25893780

[B89] Li X. Han Y. Wei Y. Acharya A. Farmer A. D. Ho J. (2014). Development of an alfalfa SNP array and its use to evaluate patterns of population structure and linkage disequilibrium. *PLoS One* 9:e84329. 10.1371/journal.pone.0084329 24416217 PMC3887001

[B90] Limborg M. T. Seeb L. W. Seeb J. E. (2016). Sorting duplicated loci disentangles complexities of polyploid genomes masked by genotyping by sequencing. *Mol. Ecol.* 25 2117–2129. 10.1111/mec.13601 26939067

[B91] Maenhout S. (2018). *Progeno.* Gent: Ghent University.

[B92] Mamanova L. Coffey A. J. Scott C. E. Kozarewa I. Turner E. H. Kumar A. (2010). Target-enrichment strategies for next-generation sequencing. *Nat. Methods* 7 111–118. 10.1038/nmeth.1419 20111037

[B93] Mason A. S. (2015). “Challenges of genotyping polyploid species,” in *Plant Genotyping: Methods and Protocols* ed. Batley J. (New York, NY: Springer) 161–168. 10.1007/978-1-4939-1966-6_12 25373756

[B94] Mason A. S. Higgins E. E. Snowdon R. J. Batley J. Stein A. Werner C. (2017). A user guide to the Brassica 60K Illumina Infinium^TM^ SNP genotyping array. *Theor. Appl. Genet.* 130 621–633. 10.1007/s00122-016-2849-1 28220206

[B95] Mather K. (1935). Reductional and equational separation of the chromosomes in bivalents and multivalents. *J. Genet.* 30 53–78. 10.1007/BF02982205

[B96] McAllister C. A. Miller A. J. (2016). Single nucleotide polymorphism discovery via genotyping by sequencing to assess population genetic structure and recurrent polyploidization in *Andropogon gerardii*. *Am. J. Bot.* 103 1314–1325. 10.3732/ajb.1600146 27466055

[B97] McCallum S. Graham J. Jorgensen L. Rowland L. J. Bassil N. V. Hancock J. F. (2016). Construction of a SNP and SSR linkage map in autotetraploid blueberry using genotyping by sequencing. *Mol. Breed.* 36:41 10.1007/s11032-016-0443-5

[B98] McCamy P. Holloway H. Yu X. Dunne J. C. Schwartz B. M. Patton A. J. (2018). A SNP-based high-density linkage map of zoysiagrass (*Zoysia japonica* Steud.) and its use for the identification of QTL associated with winter hardiness. *Mol. Breed.* 38:10 10.1007/s11032-017-0763-0

[B99] Mendel J. G. (1866). “Versuche über pflanzenhybriden,” in *Verhandlungen des Naturforschenden Vereines in Brünn Bd*. IV Abhandlungen 3–47.

[B100] Meuwissen T. H. E. Hayes B. J. Goddard M. E. (2001). Prediction of total genetic value using genome-wide dense marker maps. *Genetics* 157 1819–1829. 11290733 10.1093/genetics/157.4.1819 PMC1461589

[B101] Moncada M. D. P. Tovar E. Montoya J. C. González A. Spindel J. Mccouch S. (2016). A genetic linkage map of coffee (*Coffea arabica* L.) and QTL for yield, plant height, and bean size. *Tree Genet. Genomes* 12 1–17. 10.1007/s11295-015-0927-1

[B102] Monroe J. G. Allen Z. A. Tanger P. Mullen J. L. Lovell J. T. Moyers B. T. (2017). TSPmap, a tool making use of traveling salesperson problem solvers in the efficient and accurate construction of high-density genetic linkage maps. *BioData Min.* 10:38. 10.1186/s13040-017-0158-0 29270228 PMC5735504

[B103] Moragues M. Comadran J. Waugh R. Milne I. Flavell A. Russell J. R. (2010). Effects of ascertainment bias and marker number on estimations of barley diversity from high-throughput SNP genotype data. *Theor. Appl. Genet.* 120 1525–1534. 10.1007/s00122-010-1273-1 20157694

[B104] Motazedi E. Finkers R. Maliepaard C. De Ridder D. (2017). Exploiting next-generation sequencing to solve the haplotyping puzzle in polyploids: a simulation study. *Br. Bioinformat.* 10.1093/bib/bbw126 [Epub ahead of print]. 28065918

[B105] Neigenfind J. Gyetvai G. Basekow R. Diehl S. Achenbach U. Gebhardt C. (2008). Haplotype inference from unphased SNP data in heterozygous polyploids based on SAT. *BMC Genomics* 9:356. 10.1186/1471-2164-9-356 18667059 PMC2566320

[B106] Ng S. B. Turner E. H. Robertson P. D. Flygare S. D. Bigham A. W. Lee C. (2009). Targeted capture and massively parallel sequencing of 12 human exomes. *Nature* 461 272–276. 10.1038/nature08250 19684571 PMC2844771

[B107] Ott J. Wang J. Leal S. M. (2015). Genetic linkage analysis in the age of whole-genome sequencing. *Nat. Rev. Genet.* 16 275–284. 10.1038/nrg3908 25824869 PMC4440411

[B108] Pandey M. K. Agarwal G. Kale S. M. Clevenger J. Nayak S. N. Sriswathi M. (2017). Development and evaluation of a high density genotyping ‘Axiom_Arachis’ array with 58K SNPs for accelerating genetics and breeding in groundnut. *Sci. Rep.* 7:40577. 10.1038/srep40577 28091575 PMC5238394

[B109] Peace C. Bassil N. Main D. Ficklin S. Rosyara U. R. Stegmeir T. (2012). Development and evaluation of a genome-wide 6K SNP array for diploid sweet cherry and tetraploid sour cherry. *PLoS One* 7:e48305. 10.1371/journal.pone.0048305 23284615 PMC3527432

[B110] Piepho H.-P. Koch G. (2000). Codominant analysis of banding data from a dominant marker system by normal mixtures. *Genetics* 155 1459–1468. 10880503 10.1093/genetics/155.3.1459 PMC1461148

[B111] Plummer M. (2003). “JAGS: A. (program) for analysis of Bayesian graphical models using Gibbs sampling,” in *Proceedings of the 3rd International Workshop on Distributed Statistical Computing* Vienna 125.

[B112] Poland J. A. Brown P. J. Sorrells M. E. Jannink J.-L. (2012). Development of high-density genetic maps for barley and wheat using a novel two-enzyme genotyping-by-sequencing approach. *PLoS One* 7:e32253. 10.1371/journal.pone.0032253 22389690 PMC3289635

[B113] Pompanon F. Bonin A. Bellemain E. Taberlet P. (2005). Genotyping errors: causes, consequences and solutions. *Nat. Rev. Genet.* 6 847–859. 10.1038/nrg1707 16304600

[B114] Potato Genome Sequencing Consortium (2011). Genome sequence and analysis of the tuber crop potato. *Nature* 475 189–195. 10.1038/nature10158 21743474

[B115] Preedy K. F. Hackett C. A. (2016). A rapid marker ordering approach for high-density genetic linkage maps in experimental autotetraploid populations using multidimensional scaling. *Theor. Appl. Genet.* 129 2117–2132. 10.1007/s00122-016-2761-8 27502200

[B116] R Core Team (2016). *R: A Language and Environment for Statistical Computing.* Vienna: R Foundation for Statistical Computing.

[B117] Reddy U. K. Nimmakayala P. Abburi V. L. Reddy C. Saminathan T. Percy R. G. (2017). Genome-wide divergence, haplotype distribution and population demographic histories for Gossypium hirsutum and *Gossypium barbadense* as revealed by genome-anchored SNPs. *Sci. Rep.* 7:41285. 10.1038/srep41285 28128280 PMC5269598

[B118] Rosyara U. R. De Jong W. S. Douches D. S. Endelman J. B. (2016). Software for genome-wide association studies in autopolyploids and its application to potato. *Plant Genome* 9 1–10. 10.3835/plantgenome2015.08.0073 27898814

[B119] Rothfels C. J. Pryer K. M. Li F. W. (2017). Next-generation polyploid phylogenetics: rapid resolution of hybrid polyploid complexes using PacBio single-molecule sequencing. *New Phytol.* 213 413–429. 10.1111/nph.14111 27463214

[B120] Schmitz Carley C. A. Coombs J. J. Douches D. S. Bethke P. C. Palta J. P. Novy R. G. (2017). Automated tetraploid genotype calling by hierarchical clustering. *Theor. Appl. Genet.* 130 717–726. 10.1007/s00122-016-2845-5 28070610

[B121] Seeb J. Carvalho G. Hauser L. Naish K. Roberts S. Seeb L. (2011). Single-nucleotide polymorphism (SNP) discovery and applications of SNP genotyping in nonmodel organisms. *Mol. Ecol. Resour.* 11 1–8. 10.1111/j.1755-0998.2010.02979.x 21429158

[B122] Serang O. Mollinari M. Garcia A. A. F. (2012). Efficient exact maximum a posteriori computation for bayesian SNP genotyping in polyploids. *PLoS One* 7:e30906. 10.1371/journal.pone.0030906 22363513 PMC3281906

[B123] Shen J. Li Z. Chen J. Song Z. Zhou Z. Shi Y. (2016). SHEsisPlus, a toolset for genetic studies on polyploid species. *Sci. Rep.* 6:24095. 10.1038/srep24095 27048905 PMC4822172

[B124] Shirasawa K. Tanaka M. Takahata Y. Ma D. Cao Q. Liu Q. (2017). A high-density SNP genetic map consisting of a complete set of homologous groups in autohexaploid sweetpotato (*Ipomoea batatas*). *Sci. Rep.* 7:44207. 10.1038/srep44207 28281636 PMC5345071

[B125] Shulaev V. Sargent D. J. Crowhurst R. N. Mockler T. C. Folkerts O. Delcher A. L. (2011). The genome of woodland strawberry (*Fragaria vesca*). *Nat. Genet.* 43 109–116. 10.1038/ng.740 21186353 PMC3326587

[B126] Slater A. T. Cogan N. O. Forster J. W. Hayes B. J. Daetwyler H. D. (2016). Improving genetic gain with genomic selection in autotetraploid potato. *Plant Genome* 9 1–15. 10.3835/plantgenome2016.02.0021 27902807

[B127] Slater A. T. Wilson G. M. Cogan N. O. Forster J. W. Hayes B. J. (2014). Improving the analysis of low heritability complex traits for enhanced genetic gain in potato. *Theor. Appl. Genet.* 127 809–820. 10.1007/s00122-013-2258-7 24374468

[B128] Soltis D. E. Visger C. J. Marchant D. B. Soltis P. S. (2016). Polyploidy: pitfalls and paths to a paradigm. *Am. J. Bot.* 103 1146–1166. 10.3732/ajb.1500501 27234228

[B129] Soltis P. S. Soltis D. E. (2000). The role of genetic and genomic attributes in the success of polyploids. *Proc. Natl. Acad. Sci. U.S.A.* 97 7051–7057. 10.1073/pnas.97.13.7051 10860970 PMC34383

[B130] Spindel J. Wright M. Chen C. Cobb J. Gage J. Harrington S. (2013). Bridging the genotyping gap: using genotyping by sequencing (GBS) to add high-density SNP markers and new value to traditional bi-parental mapping and breeding populations. *Theor. Appl. Genet.* 126 2699–2716. 10.1007/s00122-013-2166-x 23918062

[B131] Stam P. (1993). Construction of integrated genetic linkage maps by means of a new computer package: join Map. *Plant J.* 3 739–744. 10.1111/j.1365-313X.1993.00739.x

[B132] Stebbins G. L. (1947). Types of polyploids: their classification and significance. *Adv. Genet.* 1 403–429. 20259289 10.1016/s0065-2660(08)60490-3

[B133] Stringham H. M. Boehnke M. (1996). Identifying marker typing incompatibilities in linkage analysis. *Am. J. Hum. Genet.* 59 946–950. 8808612 PMC1914788

[B134] Sturtevant A. H. (1913). The linear arrangement of six sex-linked factors in *Drosophila*, as shown by their mode of association. *J. Exp. Zool. Part A Ecol. Genet. Physiol.* 14 43–59. 10.1002/jez.1400140104

[B135] Su S.-Y. White J. Balding D. J. Coin L. J. (2008). Inference of haplotypic phase and missing genotypes in polyploid organisms and variable copy number genomic regions. *BMC Bioinformat.* 9:513. 10.1186/1471-2105-9-513 19046436 PMC2647950

[B136] Sukumaran S. Dreisigacker S. Lopes M. Chavez P. Reynolds M. P. (2015). Genome-wide association study for grain yield and related traits in an elite spring wheat population grown in temperate irrigated environments. *Theor. Appl. Genet.* 128 353–363. 10.1007/s00122-014-2435-3 25490985

[B137] Sverrisdóttir E. Byrne S. Sundmark E. H. R. Johnsen H. Ø. Kirk H. G. Asp T. (2017). Genomic prediction of starch content and chipping quality in tetraploid potato using genotyping-by-sequencing. *Theor. Appl. Genet.* 130 2091–2108. 10.1007/s00122-017-2944-y 28707250 PMC5606954

[B138] Swaminathan M. S. Howard H. (1953). Cytology and genetics of the potato (*Solanum tuberosum*) and related species. *Bibliogr. Genet.* 16 1–192.

[B139] Tang H. Krishnakumar V. Bidwell S. Rosen B. Chan A. Zhou S. (2014). An improved genome release (version Mt4. 0) for the model legume *Medicago truncatula*. *BMC Genomics* 15:312. 10.1186/1471-2164-15-312 24767513 PMC4234490

[B140] Tettelin H. Masignani V. Cieslewicz M. J. Donati C. Medini D. Ward N. L. (2005). Genome analysis of multiple pathogenic isolates of *Streptococcus agalactiae*: implications for the microbial “pan-genome”. *Proc. Natl. Acad. Sci. U.S.A.* 102 13950–13955. 10.1073/pnas.0506758102 16172379 PMC1216834

[B141] Tinker N. A. Bekele W. A. Hattori J. (2016). Haplotag: software for haplotype-based genotyping-by-sequencing analysis. *G*3 6 857–863. 10.1534/g3.115.024596 26818073 PMC4825656

[B142] Tinker N. A. Chao S. Lazo G. R. Oliver R. E. Huang Y.-F. Poland J. A. (2014). A SNP genotyping array for hexaploid oat. *Plant Genome* 7 1–8. 10.3835/plantgenome2015.10.0102 27898818

[B143] Tumino G. Voorrips R. E. Morcia C. Ghizzoni R. Germeier C. U. Paulo M.-J. (2017). Genome-wide association analysis for lodging tolerance and plant height in a diverse European hexaploid oat collection. *Euphytica* 213:163 10.1007/s10681-017-1939-8

[B144] Tumino G. Voorrips R. E. Rizza F. Badeck F. W. Morcia C. Ghizzoni R. (2016). Population structure and genome-wide association analysis for frost tolerance in oat using continuous SNP array signal intensity ratios. *Theor. Appl. Genet.* 129 1711–1724. 10.1007/s00122-016-2734-y 27318699 PMC4983288

[B145] Uitdewilligen J. G. Wolters A.-M. A. D’hoop B. B. Borm T. J. Visser R. G. Van Eck H. J. (2013). A next-generation sequencing method for genotyping-by-sequencing of highly heterozygous autotetraploid potato. *PLoS One* 8:e62355. 10.1371/journal.pone.0062355 23667470 PMC3648547

[B146] van Dijk T. Noordijk Y. Dubos T. Bink M. C. Meulenbroek B. J. Visser R. G. (2012). Microsatellite allele dose and configuration establishment (MADCE): an integrated approach for genetic studies in allopolyploids. *BMC Plant Biol.* 12:25. 10.1186/1471-2229-12-25 22340438 PMC3338383

[B147] van Eeuwijk F. A. Bink M. C. Chenu K. Chapman S. C. (2010). Detection and use of QTL for complex traits in multiple environments. *Curr. Opin. Plant Biol.* 13 193–205. 10.1016/j.pbi.2010.01.001 20137999

[B148] van Geest G. Bourke P. M. Voorrips R. E. Marasek-Ciolakowska A. Liao Y. Post A. (2017a). An ultra-dense integrated linkage map for hexaploid chrysanthemum enables multi-allelic QTL analysis. *Theor. Appl. Genet.* 130 2527–2541. 10.1007/s00122-017-2974-5 28852802 PMC5668331

[B149] van Geest G. Voorrips R. E. Esselink D. Post A. Visser R. G. Arens P. (2017b). Conclusive evidence for hexasomic inheritance in chrysanthemum based on analysis of a 183 k SNP array. *BMC Genomics* 18:585. 10.1186/s12864-017-4003-0 28784083 PMC5547472

[B150] Van Ooijen J. W. (2006). *JoinMap^®^ 4 Software for the Calculation of Genetic Linkage Maps in Experimental Populations.* Wageningen: Kyazma B.V.

[B151] VanRaden P. M. (2008). Efficient methods to compute genomic predictions. *J. Dairy Sci.* 91 4414–4423. 10.3168/jds.2007-0980 18946147

[B152] Voorrips R. E. Gort G. Vosman B. (2011). Genotype calling in tetraploid species from bi-allelic marker data using mixture models. *BMC Bioinformatics* 12:172. 10.1186/1471-2105-12-172 21595880 PMC3121645

[B153] Voorrips R. E. Maliepaard C. A. (2012). The simulation of meiosis in diploid and tetraploid organisms using various genetic models. *BMC Bioinformatics* 13:248. 10.1186/1471-2105-13-248 23013469 PMC3542247

[B154] Vos P. G. Paulo M. J. Voorrips R. E. Visser R. G. Van Eck H. J. Van Eeuwijk F. A. (2017). Evaluation of LD decay and various LD-decay estimators in simulated and SNP-array data of tetraploid potato. *Theor. Appl. Genet.* 130 123–135. 10.1007/s00122-016-2798-8 27699464 PMC5214954

[B155] Vos P. G. Uitdewilligen J. G. Voorrips R. E. Visser R. G. F. Van Eck H. J. (2015). Development and analysis of a 20K SNP array for potato (*Solanum tuberosum*): an insight into the breeding history. *Theor. Appl. Genet.* 128 2387–2401. 10.1007/s00122-015-2593-y 26263902 PMC4636999

[B156] VSN International (2018). *ASreml.* Hempstead: VSN International Ltd.

[B157] Vukosavljev M. Arens P. Voorrips R. Van ’t Westende W. Esselink G. D. Bourke P. M. (2016). High-density SNP-based genetic maps for the parents of an outcrossed and a selfed tetraploid garden rose cross, inferred from admixed progeny using the 68k rose SNP array. *Hortic. Res.* 3:16052. 10.1038/hortres.2016.52 27818777 PMC5080978

[B158] Wang S. Wong D. Forrest K. Allen A. Chao S. Huang B. E. (2014). Characterization of polyploid wheat genomic diversity using a high-density 90 000 single nucleotide polymorphism array. *Plant Biotechnol. J.* 12 787–796. 10.1111/pbi.12183 24646323 PMC4265271

[B159] Winfield M. O. Allen A. M. Burridge A. J. Barker G. L. Benbow H. R. Wilkinson P. A. (2016). High-density SNP genotyping array for hexaploid wheat and its secondary and tertiary gene pool. *Plant Biotechnol. J.* 14 1195–1206. 10.1111/pbi.12485 26466852 PMC4950041

[B160] Yang J. Moeinzadeh M.-H. Kuhl H. Helmuth J. Xiao P. Haas S. (2017a). Haplotype-resolved sweet potato genome traces back its hexaploidization history. *Nat. Plants* 3 696–703. 10.1038/s41477-017-0002-z 28827752

[B161] Yang X. Sood S. Glynn N. Islam M. S. Comstock J. Wang J. (2017b). Constructing high-density genetic maps for polyploid sugarcane (*Saccharum* spp.) and identifying quantitative trait loci controlling brown rust resistance. *Mol. Breed.* 37:116 10.1007/s11032-017-0716-7

[B162] You Q. Yang X. Peng Z. Xu L. Wang J. (2018). Development and applications of a high throughput genotyping tool for polyploid crops: single nucleotide polymorphism (SNP) array. *Front. Plant Sci.* 9:104. 10.3389/fpls.2018.00104 29467780 PMC5808122

[B163] Young N. D. Debellé F. Oldroyd G. E. Geurts R. Cannon S. B. Udvardi M. K. (2011). The *Medicago* genome provides insight into the evolution of rhizobial symbioses. *Nature* 480 520–524. 10.1038/nature10625 22089132 PMC3272368

[B164] Yu J. Pressoir G. Briggs W. H. Vroh Bi I. Yamasaki M. Doebley J. F. (2006). A unified mixed-model method for association mapping that accounts for multiple levels of relatedness. *Nat. Genet.* 38 203–208. 10.1038/ng1702 16380716

[B165] Yu L. X. Zheng P. Zhang T. Rodringuez J. Main D. (2017). Genotyping-by-sequencing-based genome-wide association studies on *Verticillium* wilt resistance in autotetraploid alfalfa (*Medicago sativa* L.). *Mol. Plant Pathol.* 18 187–194. 10.1111/mpp.12389 26933934 PMC6638244

[B166] Zhang K. Calabrese P. Nordborg M. Sun F. (2002). Haplotype block structure and its applications to association studies: power and study designs. *Am. J. Hum. Genet.* 71 1386–1394. 12439824 10.1086/344780 PMC378580

[B167] Zhang T. Yu L.-X. Zheng P. Li Y. Rivera M. Main D. (2015). Identification of loci associated with drought resistance traits in heterozygous autotetraploid alfalfa (*Medicago sativa* L.) using genome-wide association studies with genotyping by sequencing. *PLoS One* 10:e0138931. 10.1371/journal.pone.0138931 26406473 PMC4583413

[B168] Zhang Z. Ersoz E. Lai C.-Q. Todhunter R. J. Tiwari H. K. Gore M. A. (2010). Mixed linear model approach adapted for genome-wide association studies. *Nat. Genet.* 42 355–360. 10.1038/ng.546 20208535 PMC2931336

[B169] Zheng C. Voorrips R. E. Jansen J. Hackett C. A. Ho J. Bink M. C. (2016). Probabilistic multilocus haplotype reconstruction in outcrossing tetraploids. *Genetics* 203 119–131. 10.1534/genetics.115.185579 26920758 PMC4858767

